# Resolution of the ABA Biosynthesis Controversy: Discovery of a Dihydroxylating Terpene Synthase and Its Convergent Evolution

**DOI:** 10.1021/acschembio.6c00103

**Published:** 2026-05-26

**Authors:** Víctor Coca-Ruiz, Katharina Hausmann, Gerald Dräger, Henry Struwe, Maria Zahid, Josefina Aleu, Dörte Solle, Andreas Kirschning, Isidro G. Collado, Sascha Beutel

**Affiliations:** † Department of Organic Chemistry, Faculty of Sciences, University of Cádiz, Puerto Real, Cádiz 11510, Spain; ‡ Institute of Biomolecules (INBIO), University of Cádiz, Puerto Real, Cádiz 11510, Spain; § Institute of Technical Chemistry, 26555 Leibniz University of Hannover, 30167 Hannover, Germany; ∥ Institute of Organic Chemistry, Leibniz University of Hannover, 30167 Hannover, Germany; ⊥ Uppsala Biomedical Center (BMC), Uppsala University, Husargatan 3, Uppsala 752 37, Sweden

## Abstract

Fungal terpene biosynthesis is a vital source of bioactive metabolites. Here, we elucidate the function of BcStc5, a *Botrytis cinerea* terpene synthase previously linked to abscisic acid (ABA) biosynthesis. Using bioinformatics and heterologous expression, we demonstrate that BcStc5 catalyzes the formation of (*4S*,*5S*,*7R*,*10S*)-4β,10α-eudesmane-5β,11-diol (**1**), a dihydroxylated sesquiterpenoid not previously reported in fungi. Mechanistic modeling suggests BcStc5 employs a novel, P450-independent dihydroxylation strategy. We further established a scalable bioproduction platform in *Escherichia coli*, achieving titers >550 mg/L. This work resolves a long-standing biosynthetic controversy, characterizing BcStc5 as an eudesmanediol synthase rather than the ABA cyclase, settling the controversy over this protein.

## Graphical abstract

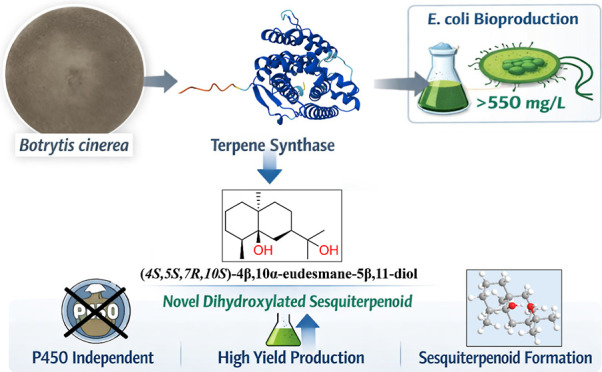

## 1 Introduction

The necrotrophic fungus *Botrytis cinerea* causes gray mold in over 1400 plant species,^1^ resulting in annual agricultural losses between 10$ billion and 100$ billion.^2^ Its aggressive infection strategy involves the secretion of secondary metabolites and lytic enzymes to induce host necrosis.^2^ Among these, sesquiterpenoids such as the phytotoxin botrydial act as essential virulence factors.^3^ Decoding their biosynthesis is crucial to understanding the molecular interplay between pathogen and host.

In this context, fungi are a prolific source of terpenoids,^1^ which serve as chemical signals and defense agents in ecological interactions.^4^ Terpene synthases (TPSs) are the gatekeepers of this diversity, cyclizing precursors like farnesyl diphosphate (FDP) via complex carbocation cascades.^4^ Class I TPSs, such as BcStc5, initiate catalysis through Mg^2+^-promoted ionization of the diphosphate group, triggering a cyclization sequence that defines the carbon scaffold.^5^

Following this initial cyclization, terpenoid diversification canonically involves a division of labor: a TPS constructs the hydrophobic scaffold, which is subsequently decorated with hydroxyl groups by cytochrome P450 monooxygenases (P450s).^6^ This paradigm—where TPS dictates the skeleton and P450 the oxidation pattern—has fueled significant controversy regarding the biosynthesis of abscisic acid (ABA) in *B. cinerea*.

This controversy is rooted in the fact that unlike plants, fungi synthesize the hormone ABA directly from FDP.^7^ However, the identity of the initial cyclase is disputed. Izquierdo-Bueno et al. proposed the Class I TPS BcStc5 (BcAba5) as the key enzyme, as Δ*Bcstc5* mutants abolished ABA production.^7^ Conversely, subsequent work identified BcAba3—a novel, nonhomologous enzyme—as the true cyclase.^8–10^ This discrepancy creates a fundamental question: if BcAba3 is the cyclase, why does deleting *Bcstc5* abolish production? This article resolves the controversy by characterizing BcStc5 as an eudesmanediol synthase, demonstrating that its product, eudesmane-5β,11-diol (**1**), is the molecule responsible for the previously observed phenotypes.

## 2 Results

### 2.1 A Widespread and Syntenically Conserved Biosynthetic Gene Cluster

A comprehensive bioinformatic survey of fungal genomes identified a previously uncharacterized and highly conserved biosynthetic gene cluster (BGC) across diverse genera within the *Ascomycota*. The core of this BGC consists of a physically colocalized gene pair: one encoding a putative terpene synthase (TS) containing a “Terpene synthase family 2” domain (Pfam: PF19086) and the other encoding a cytochrome P450 (P450) of the PF00067 Pfam family. This conserved genomic architecture was observed not only in multiple phytopathogenic strains of *B. cinerea* and related *Botrytis* species but also extended to other genera within the *Sclerotiniaceae*, *Nectriaceae*, and *Aspergillaceae* families, including *Sclerotinia*, *Fusarium*, and *Penicillium*.^11^

The persistent physical proximity of the TS and P450 genes—often separated by less than one kilobase—suggests a strong selective pressure for coregulation and implies an obligatory functional link between the two enzymes. This arrangement, where the P450 likely acts as a dedicated tailoring enzyme for the TS-generated hydrocarbon scaffold, led to the central hypothesis that the BGC directs the biosynthesis of an oxidized sesquiterpenoid.^11^ The recurrence of this specific BGC across evolutionarily distant phytopathogenic fungi suggests it may function as a conserved pathogenic toolkit.^12^

Furthermore, the BGC exhibits architectural plasticity across different species, pointing to functional adaptation and modular evolution. For instance, the clusters in *Thelonectria olida* and *Fusarium decemcellulare* include a gene for a Major Facilitator Superfamily (MFS) transporter, suggesting the final metabolite is actively exported from the cell to function in the extracellular environment. In *Penicillium samsorium*, the core TS/P450 pair is flanked by peptide-modifying enzymes, implying the production of a complex hybrid sesquiterpenoid-peptide metabolite. These variations suggest a model where a core TS/P450 chassis is acquired by different fungal lineages and subsequently upgraded with accessory genes tailored to a specific ecological niche. The complete architectural comparison of the gene cluster between strains is detailed in Figure 1.

**Figure 1:**
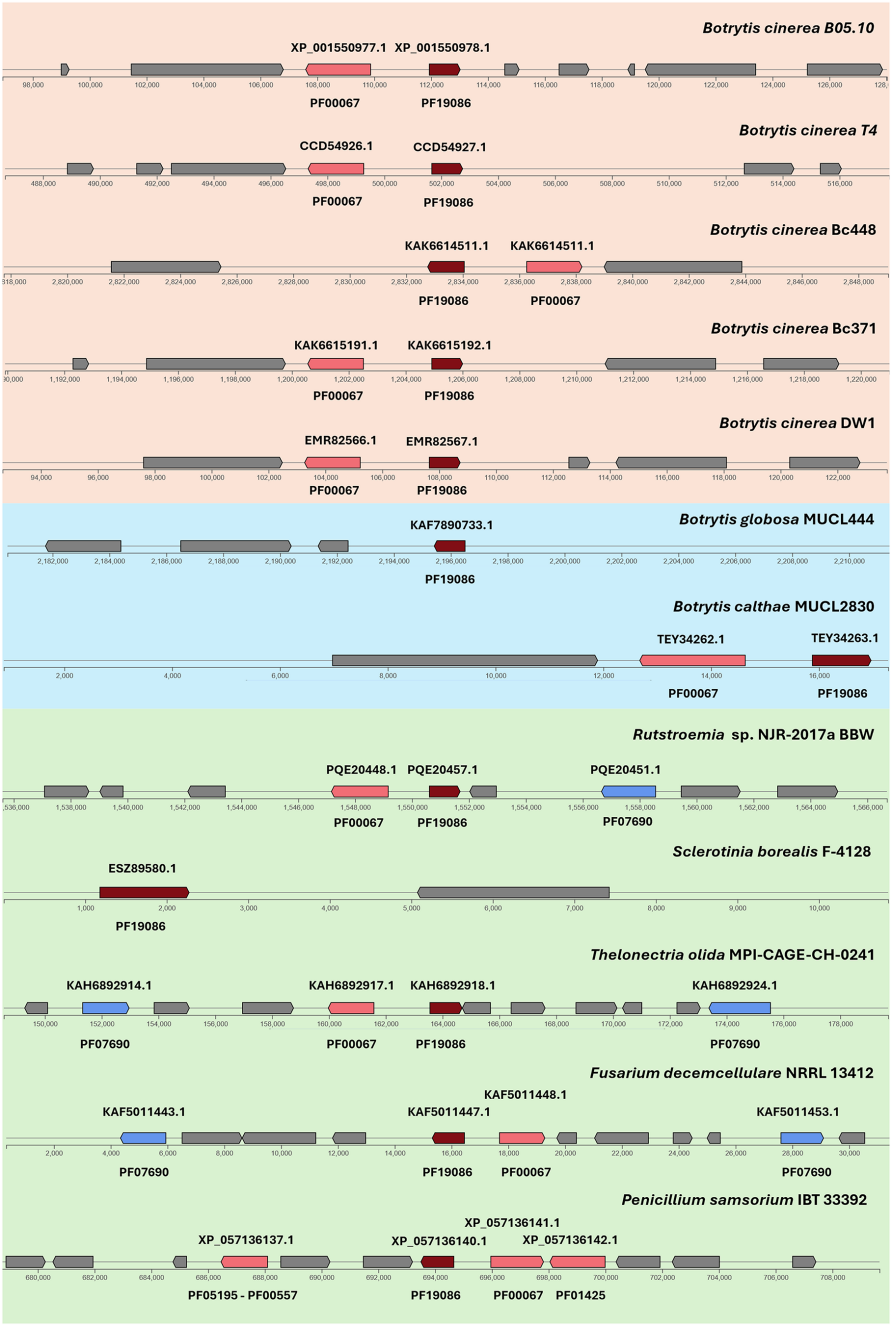
Genomic organization of the conserved sesquiterpenoid biosynthetic gene clusters across different fungal species. The core gene Terpene synthase family 2 (PF19086) is indicated in red, the Major Facilitator Superfamily (PF07690) in blue, and additional biosynthetic genes, including Cytochrome P450 (PF00067), Amidase (PF01425), Aminopeptidase (PF05195), and Metallopeptidase family M24 (PF00557), are indicated in pink.

### 2.2 The Core Terpene Synthase BcStc5 has a Patchy Phylogenetic Distribution

To elucidate the evolutionary trajectory of the terpene synthase associated with this cluster, we performed a homology analysis of the *BcStc5* gene across the *Botrytis* genus (Table S1). This analysis revealed discontinuous phylogenetic distribution (Note S1); homologues of *BcStc5* are strictly conserved in polyphagous species such as *B. cinerea* and *Botrytis pseudocinerea* but are notably absent from numerous host-specialist species.

This strict correlation with a generalist pathogenic lifestyle marks *BcStc5* as an accessory gene that likely confers a selective advantage for infecting a broad range of hosts, rather than a core gene essential for fundamental fungal viability.^12^ A broader phylogenetic analysis placed BcStc5 within a well-supported clade of terpene synthases from the *Sclerotiniaceae* family, with close homologues in *Rutstroemia* and *Sclerotinia* species (74–85% identity). More distant homologues were identified across other Ascomycota classes and even in the phylum Basidiomycota, albeit with lower sequence identity (Table S2).

The combination of a patchy distribution within its own genus and the presence of orthologs in both closely and distantly related fungi is inconsistent with a simple model of vertical inheritance and differential gene loss.^12^ A more parsimonious explanation is the acquisition of *BcStc5* by the ancestral lineage of generalist *Botrytis* species via Horizontal Gene Transfer (HGT). The retention of this gene only in lineages where it confers an adaptive advantage suggests its product provides a tangible benefit for overcoming the diverse defenses of multiple plant hosts. This putative HGT implies that pathogenic capabilities can be acquired as modular functional units, a mechanism for the rapid evolution of virulence with significant implications for agriculture and disease management.

### 2.3 Transcriptional Divergence from Core Virulence Factors

To further investigate the putative ecological role of BcStc5, we analyzed its transcriptional regulation in comparison to *BcBot2* (Bcin12g06390), the well-characterized sesquiterpene synthase responsible for producing the phytotoxin botrydial, a primary virulence factor in *B. cinerea*. Using a comprehensive set of publicly available RNA-Seq data curated in the *B. cinerea* Gene Expression Browser (BEB),^13^ we compared the expression profiles of both genes across dozens of experimental conditions (Figure S1). These included vegetative growth on various culture media (*in vitro*) and during the infection of diverse plant hosts such as tomato (*Solanum lycopersicum*), grape (*Vitis vinifera*), and *Arabidopsis thaliana* (*in planta*).

The analysis revealed different expression patterns between the two genes, supporting distinct biological functions. As expected for a primary virulence factor, *BcBot2* showed low basal expression during growth on standard nutrient-rich media but was significantly upregulated during host infection.^13,14^ This host-induced expression is consistent with the deployment of botrydial to kill plant tissue and facilitate necrotrophic colonization.

In striking contrast, *BcStc5* (Bcin01g03520) exhibited a markedly different transcriptional profile. It displayed a more constitutive and moderate level of expression across both *in vitro* and *in planta* conditions. Crucially, its expression was not specifically induced during the initial stages of plant infection in the same manner as *Bcbot2*. This pattern suggests that the product we identified as eudesmanediol (**1**) is not deployed as a primary virulence factor. Instead, its broader expression profile is more consistent with a role in general fitness, defense, and ecological competence.^15^ This function is consistent with with the potent antimicrobial activity of its product, which would serve to inhibit competing microbes encountered on the host plant surface or in the surrounding environment, thereby securing the niche for a generalist pathogen like *B. cinerea*. These divergent expression profiles provide strong transcriptomic evidence that BcStc5 does not function as a classical virulence factor but rather contributes to the broader ecological success of the fungus.

### 2.4 In Silico Analyses Define a Mechanistic Framework

To formulate a mechanistic hypothesis for BcStc5 function, we conducted detailed sequence and structural analyses. A MEME analysis of 274 orthologous sequences identified three highly conserved motifs (Figure S2). Motif 1 corresponds to the canonical aspartate-rich **DDxxD** motif, essential for binding the Mg^2+^ cofactor and substrate diphosphate group, whereas Motif 2 matches the **(N,D)­Dxx­(S,T)­xxxE** motif, which is crucial for stabilizing carbocation intermediates.^5,16^ Both are defining hallmarks of class I terpene cyclases. Motif 3 likely contributes to the structural integrity of the active site pocket that dictates product specificity.^16^

Furthermore, a ConSurf analysis, which maps evolutionary conservation onto the BcStc5 amino acid sequence, provided a structural and functional map of the enzyme (Table S3 and Figure S3). This analysis revealed a central catalytic domain (residues ∼ 91–324) comprising extensive, highly conserved surfaces predicted to form a large, hydrophobic active site pocket, which is buttressed by a conserved structural core essential for maintaining the enzyme’s fold. This architecture is a prerequisite for binding the flexible farnesyl diphosphate (FDP) substrate, guiding its complex cyclization cascade, and stabilizing reactive intermediates.

The large volume of this conserved cavity, rather than a small, discrete active site, strongly suggests that BcStc5 facilitates a complex, multistep catalytic reaction. Specific conserved regions, such as the **DYFSWHVEKD** motif (residues 234–243), are rich in polar and aromatic residues ideally positioned for protonation (Asp235, Glu242) and carbocation stabilization via cation–π interactions (Tyr234, Phe236, Trp238).^5^ Together, these *in silico* analyses provide a robust mechanistic framework, defining BcStc5 as a canonical class I terpene cyclase.

### 2.5 BcStc5 Contains a Buried Binding Pocket for FDP

Molecular docking simulations were performed to predict the binding mode of the farnesyl z (Figure 2). The BcStc5 homology model was processed to remove steric clashes and optimize side chains. Docking results were evaluated based on predicted binding affinities (Δ*G*) and the quality of fit within the active site. Among the generated poses, the conformation with the lowest binding energy (−8.008 kcal/mol) was selected for detailed analysis and visualization.

**Figure 2:**
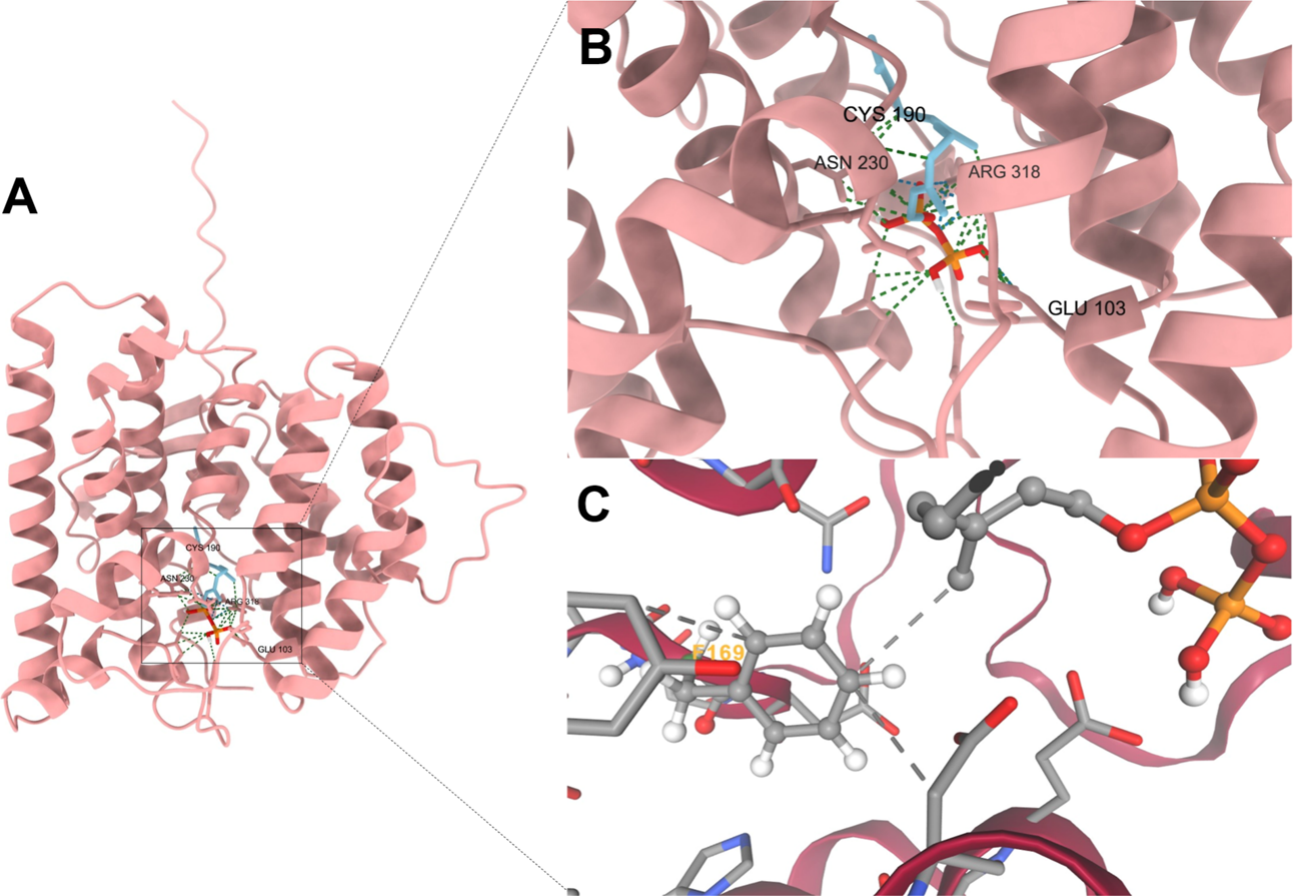
(A) The AlphaFold (AF) model of BcStc5. (B) The hydrogen bonds are shown in blue dotted line, where as ionic interactions are shown in green dotted lines. (C) The hydrophobic interaction with PHE169 is shown in gray lines.

Key molecular interactions were identified between the FDP and BcStc5 residues. Hydrogen bonds were observed with **Glu103**, **Asn230**, **and Arg318**, while additional ionic contacts involved **Glu103, Cys190, Glu238, and Gln241**. A hydrophobic interaction with **Phe169** further stabilizes the structure. Visualization and interaction profiling were performed using ChimeraX.

The docking simulations provide evidence that the ligand exhibits favorable binding within the active site of BcStc5, with predicted binding energies in the range of −7.7 to −8.0 kcal/mol (Table S4). The narrow distribution of affinities across the top-ranked poses indicates strong binding and supports the reliability of predictions. Key interactions, including hydrogen bonds with Glu103, Asn230, and Arg318, as well as hydrophobic contacts with Phe169, are consistent with residues previously implicated in substrate recognition and stabilization in related enzymes. These findings suggest that the ligand is likely to occupy a physiologically relevant binding mode, reinforced by the observed ionic interactions with Cys190, Glu238, and Gln241. Among these residues, Glu103, Asn230, Arg318, Glu238, and Gln241 are highly conserved. While computational docking cannot fully capture protein flexibility or solvent effects, the consistency of binding energies and biologically plausible interaction network provide confidence in the predicted binding modes.

### 2.6 BcStc5 Functions as a Hub Within a Metabolic Supercluster

To define the functional context of BcStc5, we computationally predicted the protein–protein interaction (PPI) network for a set of 11 biosynthetic enzymes from *B. cinerea* strain T4, including BcStc5 (protein ID: G2YT23) (Figure 3). The analysis revealed that these proteins form a single, cohesive network with a high average node degree of 8.55 and high-confidence interaction scores (Table S5). This topology indicates a highly coordinated functional unit, or metabolic supercluster, rather than a simple linear pathway.

**Figure 3:**
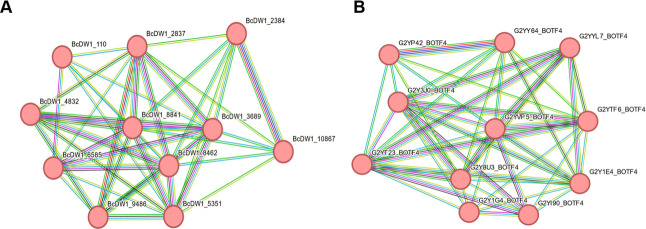
Protein–protein interaction networks of the metabolic supercluster in *B. cinerea* for *B. cinerea* (A) Dw1 and (B) T4.

Gene Ontology (GO) enrichment analysis confirmed that the supercluster is centered on isoprenoid and terpenoid biosynthesis (FDR < 1.0 × 10^–9^) (Table S6 and Figure S4). Strikingly, the network integrates two additional functional modules: one for protein prenylation (farnesyltransferase subunits G2Y1G4 and G2YI90) and another for tryptophan biosynthesis (G2YP42 and G2YY64). The high-confidence interactions linking these modules to the terpenoid core establish a terpenoid–tryptophan axis, suggesting a sophisticated mechanism for coregulation or substrate channeling.^17^

This discovery challenges the classic secondary-metabolism paradigm of discrete BGCs by proposing metabolic superclusters as a higher level of organization involving the functional association of entire pathways. (Further details on the supercluster organization are in Note S2). This architecture could enable efficient substrate channeling of FDP, coregulation of disparate pathways, and the synthesis of complex hybrid molecules by bringing distinct chemical machineries into close proximity. The terpenoid–tryptophan axis, for instance, suggests a latent capacity to produce indole-diterpenoid natural products, a finding with major implications for future genome-mining strategies. This integrated architecture is a deeply conserved feature, as a parallel analysis of homologous proteins from the reference strain *B. cinerea* DW1 revealed a nearly identical supercluster organization (Table S7). This points to a broad evolutionary strategy in fungi for generating chemical diversity by integrating distinct metabolic pathways.^18^

### 2.7 Heterologous Expression Reveals BcStc5 Produces a Dihydroxylated Eudesmanoid

To determine the function of BcStc5, two approaches were used. The sequence of the *BcStc5* protein was obtained from the EnsemblFungi database and subsequently codon-optimized to ensure efficient expression in an *Escherichia coli* host. The enzyme was purified, and the activity of the enzyme was tested *in vitro*. In addition, the mevalonate pathway from *Saccharomyces cerevisiae* ^19^ was coexpressed with BcStc5. This ensures the supply of the substrate farnesyl diphosphate (FDP), thereby creating a whole-cell biocatalytic platform, which allows for the production of the desired sesquiterpene from simple carbon sources.^19^

Optimization of the heterologous expression system was crucial. Initial expression trials using standard conditions (37 °C induction) resulted in insoluble protein accumulation in inclusion bodies. To address this, we systematically optimized expression conditions, testing various temperatures, IPTG concentrations, and induction times (Table S8). Optimal soluble expression was achieved at 18 °C with 0.5 mM IPTG for 16 h. The protein was purified using Ni-NTA affinity chromatography followed by size-exclusion chromatography, yielding approximately 5 mg of purified protein per liter of culture with >95% purity as assessed by SDS-PAGE.

Both approaches, the *in vitro* assay as well as the whole-cell biocatalytic production, yielded one major sesquiterpenoid. GC–MS analysis indicated the product was a diol (Figure S5), and high-resolution mass spectrometry (HRMS) established a molecular formula of C_15_H_28_O_2_, with an observed [M + Na]^+^ ion at *m*/*z* 263.1986 (calculated for C_15_H_28_O_2_Na, 263.1987) (Figure S6). This result unequivocally links BcStc5 to the synthesis of a dihydroxylated sesquiterpenoid, resolving a long-standing debate by demonstrating that BcStc5 is not involved in abscisic acid (ABA) biosynthesis.^7^ The discovery that the terpene synthase alone performs a dual hydroxylation–cyclization reaction, a function typically ascribed to colocalized P450 monooxygenases, reveals a remarkable and unexpected catalytic versatility that expands the known catalytic scope of the terpene synthase family (Figure 4).

**Figure 4:**
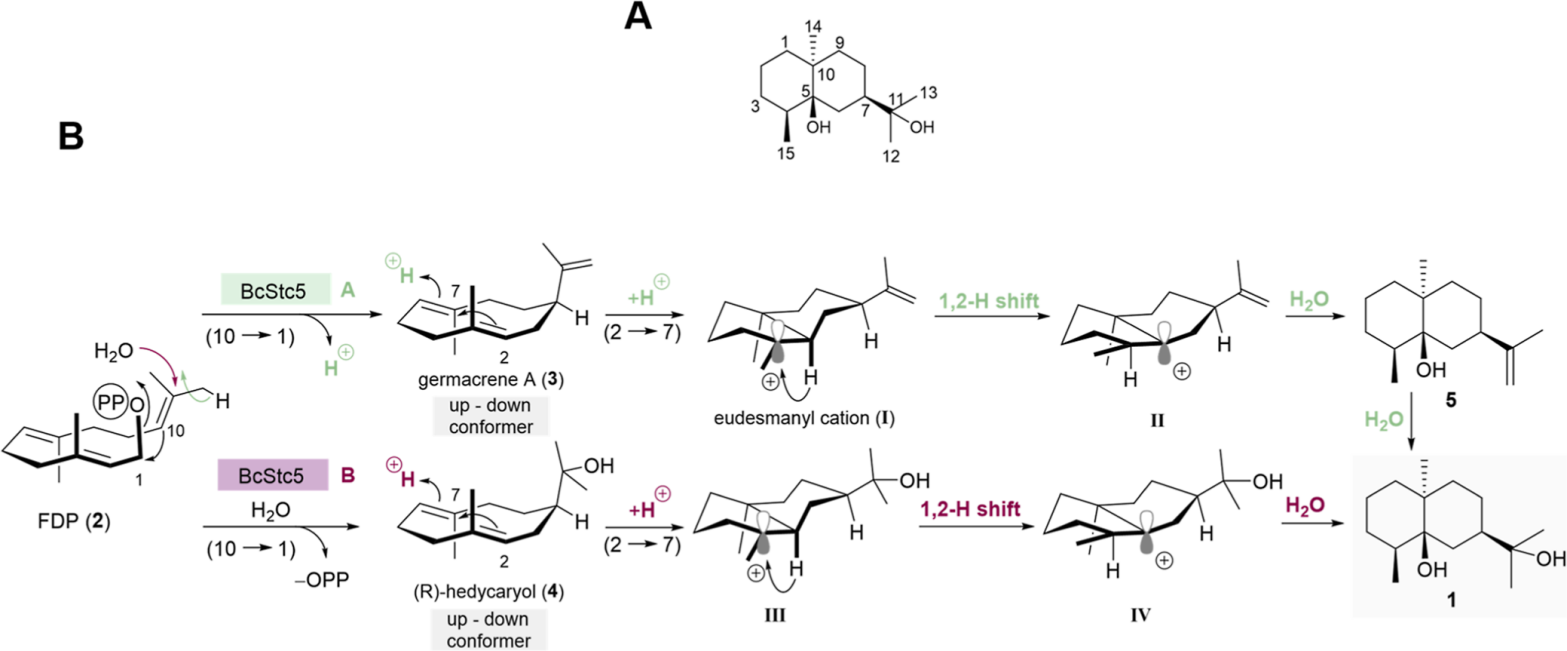
Structure and absolute stereochemistry of (*4S*,*5S*,*7R*,*10S*)-4β,10α-eudesmane-5β,11-diol (eudesmanediol) (**1**), (A) shown in 2D and (B) proposed biosynthetic pathway leading to eudesmanediol.

### 2.8 NMR and X-ray Crystallography Define the Product Structure

The structure of the isolated product was elucidated through extensive 1D and 2D NMR spectroscopy (Figures 4A and S7–S12). The ^13^C NMR spectrum Figure S8) showed 15 signals corresponding to four methyl, six methylene, two methine, and three quaternary carbons. Two downfield quaternary signals at δ73.2 and δ72.9 confirmed the presence of two hydroxyl-bearing carbons.

The complete carbon skeleton was established using 2D NMR, with key Heteronuclear Multiple Bond Correlation (HMBC) signals confirming an eudesmane-type scaffold and positioning the hydroxyl groups at C_5_ and C_11_. The ^1^H COSY spectrum allowed tracing of spin systems in the eudesmane decalin core (Figure S9). Absence of vicinal couplings for protons at C_5_ and C_11_ confirmed hydroxyl groups are attached to quaternary carbons. Detailed HMBC analysis provided critical correlations (Figure S10). Correlations from methyl protons H_12_/H_13_ to C_7_, C_11_, and C_12_ unequivocally establish the isopropylol side chain structure. Correlations from angular methyls H_14_/H_15_ to quaternary carbons C_4_/C_10_ confirm the *gem*-dimethyl substitution and eudesmane skeleton architecture. The Multiplicity-edited HSQC spectrum allowed unequivocal assignment of diastereotopic methylene protons in the rigid ring system, resolving spectral overlap in the aliphatic region (Figure S11).

To further confirm the structure and additionally establish its absolute stereochemistry (Figure S12), the new product was crystallized and analyzed by single-crystal X-ray diffraction. The crystallographic data unambiguously identified the product as (*4S*,*5S*,*7R*,*10S*)-4β,10α-eudesmane-5β,11-diol (**1**), wherein the hydroxyl protons exhibit a two-state disorder, each with 50% occupancy.

This is the first report of **1** from a fungal source. Intriguingly, compound **1**, with the same absolute configuration, has been previously isolated from the aromatic grass *Cymbopogon distans*,^20^ *Streptomyces anulatus* isolated from *Giraffa camelopardalis* feces,^21^ and a bacterial mangrove endophyte (*Streptomyces* sp. JMRC:ST027706) associated with the mangrove plant *Bruguiera gymnorrhiza*.^22,23^ This striking instance of convergent evolution across three domains of life strongly implies that **1** possesses a conserved and potent biological activity that confers a significant selective advantage. The maintenance of energetically costly metabolic pathways across such vast evolutionary distances implies strong positive selective pressure. The most parsimonious explanation for this pressure is that the molecule itself performs a valuable, conserved function in each organism’s ecological niche.

### 2.9 Proposed Mechanism for the Dual Hydroxylation-Cyclization Cascade

The formation of eudesmanediol **1** from the linear precursor FDP within a single active site necessitates a complex, multistep catalytic cascade. We propose a mechanism initiated by the ionization of FDP, followed by a canonical 1,10-cyclization from the Si face of the C_10_–C_11_ double bond to form the (*R*)-germacrenyl cation. Nucleophilic attack by a water molecule at C_11_ yields the (*R*)-hedycaryol intermediate.^16,24^ This flexible molecule is then repositioned within the active site, a conformational gating step that preorganizes it for the second phase of the reaction.^5^ Proton-induced cyclization of the hedycaryol intermediate generates a bicyclic, *cis*-fused eudesmane cation. This is followed by a stereospecific 1,2-hydride shift from C_5_ to C_4_, relocating the positive charge to C_5_. The cascade terminates with the attack of a second water molecule at the C_5_ carbocation, yielding the final product **1** with its experimentally determined stereochemistry (see Note S3 for a comprehensive mechanistic analysis).^25,26^

The cationic cascade yielding diol **1** from the linear precursor FDP (**2**) within a single active site necessitates a complex, multistep catalytic cascade. Our proposed formation of **1** commences from FDP via an initial 1,10 cyclization,^16^ followed either by a subsequent proton abstraction toward germacrene A (**3**)^24^ (pathway A) or the nucleophilic attack of water forming (*R*)-hedycaryol (**4**) (pathway B).^27,28^ If germacrene A is formed as an intermediary product, a reprotonation at C6 induces a 2,7 cyclization followed by a 1,2-hydride shift resulting in cation **II** (Figure 4B). The nucleophilic attack of water would lead to the formation of crotoscheffleriune I (**5**). The proton induced addition of water to the isopropylidene group would yield the final sesquiterpene diol **1** (pathway A) (Figure 4B). Alternatively, **4** follows an analogous sequence of protonation, cyclization and hydride shift to form cation **IV**, followed by the nucleophilic attack of water resulting in **1** (pathway B) (Figure 4B).

This catalytic strategy, which relies on chaperoning an uncharged intermediate through distinct reaction coordinates, has been established in other complex terpene synthases^29^ and provides a clear functional rationale for the large, conserved active site architecture predicted by our *in silico* analysis; such a vast catalytic surface is the structural prerequisite for accommodating this intricate, multistep transformation.^16^

### 2.10 Process Modeling Defines a Growth–Production Trade-off

To systematically optimize the whole-cell biocatalytic synthesis of eudesmanediol (**1**), we implemented a two-phase Design of Experiments (DoE) strategy. First, we performed a 2^4–1^ fractional factorial design with three central replicates (11 runs total) to screen the effects of four key variables: postinduction time (*t*), cold-shift induction temperature (Δ*T*), IPTG concentration, and glycerol concentration. The analysis of this initial screening revealed that neither IPTG nor glycerol concentration had a statistically significant impact on product yield within the tested ranges.^30^

Based on these findings, we advanced to a focused optimization targeting the two critical variables: cultivation time and induction temperature. For this, we employed a Face-Centered Central Composite Design (CCF) with 11 experimental runs, including three center point replicates. The resulting data were fitted to a partial least squares (PLS) regression model, which demonstrated outstanding quality and predictive power. The model’s robustness is confirmed by a high goodness of fit (*R* ^2^ = 0.851) and predictive capability (*Q* ^2^ = 0.497). The strong correlation shown in the Observed vs Predicted plot further validates the model’s high accuracy.^30^

Analysis of the model coefficients quantifies the impact of each variable. Cultivation time (*t*) emerged as the most influential variable with a strong positive coefficient. Crucially, significant negative interaction terms (e.g., *t* x Δ*T*) reveal a complex, nonlinear relationship, indicating that the optimal temperature profile is intrinsically linked to the cultivation duration. This interplay is best visualized in the Response Contour plot, which maps the production landscape. The plot illustrates that the highest product concentrations are achieved by combining the longest cultivation time (4 days) with a substantial temperature drop to a final 12–15 °C. Under these refined conditions, we consistently achieved product titers exceeding 550 mg/L in a 4 L flask with 1 L of culture media, thereby establishing a robust, high-yield platform for producing this bioactive sesquiterpenoid (Figure 5 and Table 1).

**Figure 5:**
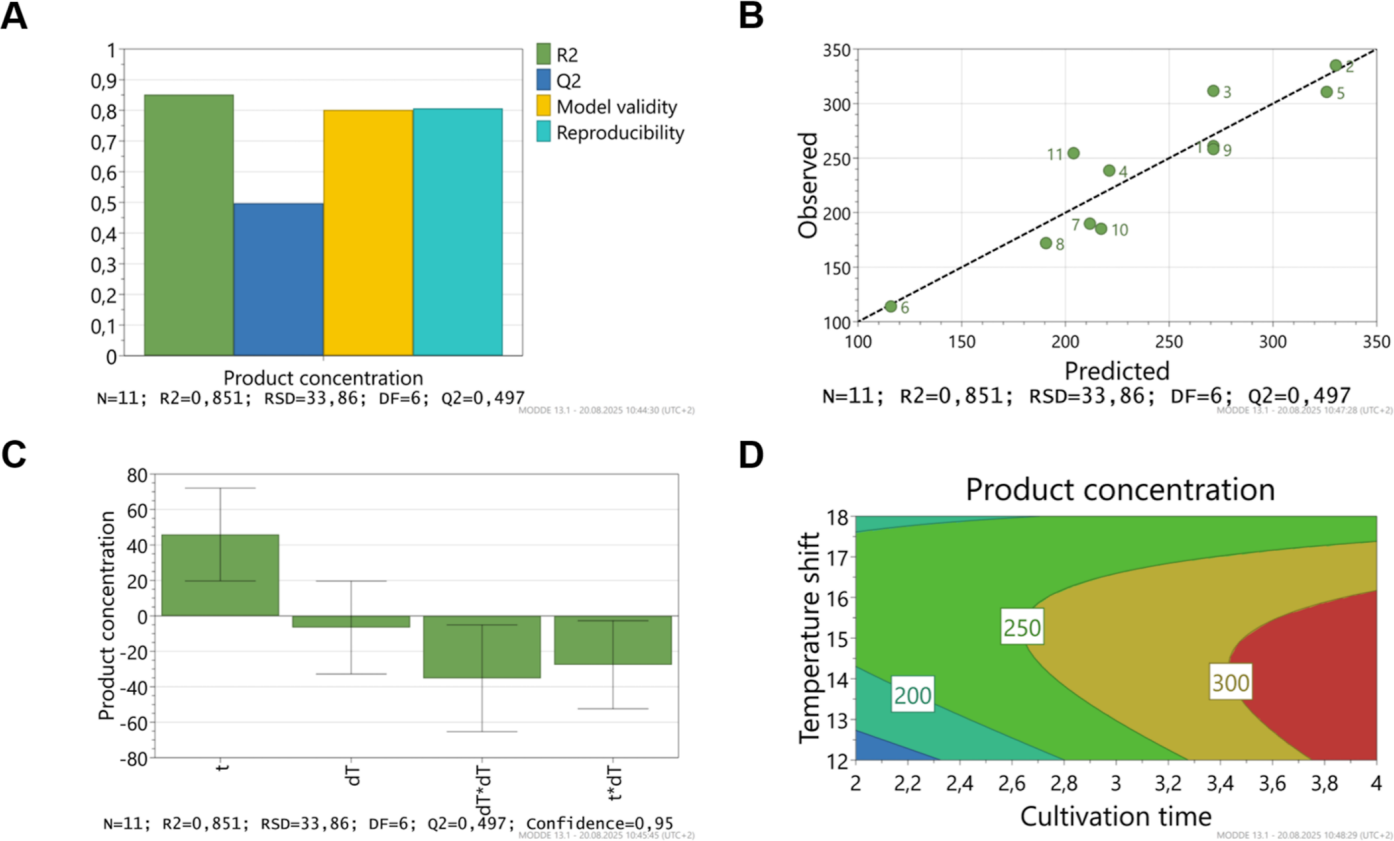
Statistical modeling and optimization of eudesmanediol biosynthesis. (A) Summary of Fit plot for the partial least squares (PLS) model. The model shows a high goodness of fit (*R* ^2^ = 0.851), good predictive power (*Q* ^2^ = 0.497), high validity, and excellent reproducibility of the experiments. (B) Observed versus predicted product concentration. The strong correlation of the 11 experimental runs validates the model’s accuracy. (C) Scaled and centered coefficient plot showing the relative impact of cultivation time (t), temperature shift (Δ*T*), and their interactions on product titer. Error bars represent the 95% confidence interval. (D) Response contour plot illustrating the predicted product concentration as a function of cultivation time and temperature shift, identifying the optimal design space for achieving high titers (>300 units, red area).

**Table 1:** Comparison of Microbial Production Titers for Selected Sesquiterpenoids

sesquiterpenoid	product class	host organism	titer (mg/L or g/L)	reference
**eudesmanediol (1)**	diol	E. coli	**>550 **mg/L****	**this work**
5-*epi*-jinkoheremol	alcohol	S. cerevisiae	875 mg/L	^31^
zerumbone	ketone	S. cerevisiae	40 mg/L	^32^
β-caryophyllene	hydrocarbon	E. coli	5.1 g/L	^33^
germacrene D	hydrocarbon	S. cerevisiae	7.9 g/L	^34^
amorphadiene	hydrocarbon	S. cerevisiae	>40 g/L	^35^
farnesene	hydrocarbon	S. cerevisiae	>14.7 g/L	^36^

## 3 Discussion

Our findings provide definitive evidence that resolves a significant and persistent controversy in the field of fungal hormones. The direct production of eudesmanediol (**1**) by BcStc5 conclusively demonstrates that this enzyme is not, as previously proposed, the key sesquiterpene cyclase responsible for the first committed step in abscisic acid (ABA) biosynthesis. On the other hand, our results strongly support the parallel findings of Takino et al. (2018, 2019) and Otto et al. (2019), who functionally reconstituted the complete ABA pathway in heterologous hosts (*Aspergillus oryzae* and *S. cerevisiae*, respectively).^8–10^ Their work demonstrated that the four genes of the canonical ABA cluster (*Bcaba1*, *Bcaba2*, *Bcaba3*, *Bcaba4*) are necessary and sufficient for ABA production. Crucially, they identified BcAba3 (a novel and structurally distinct synthase class with no homology to canonical terpene cyclases like BcStc5) as the enzyme that converts FDP to the ABA precursor, α-ionylideneethane. By providing a definitive, alternative function for BcStc5, our work removes the ambiguity and cements the role of BcAba3 as the true initiating enzyme of the ABA pathway in fungi (Table 2).

**Table 2:** Functional Comparison Resolving the ABA Biosynthesis Controversy

enzyme	proposed function	key evidence	conclusion
BcStc5 (BcAba5)	eudesmanediol synthase	heterologous expression + structural elucidation	BcStc5 is a dihydroxylating eudesmanediol synthase. Its effect on ABA is likely indirect and regulatory
BcAba3	ABA precursor synthase	heterologous pathway reconstitution	*Bcaba3* is the bona fide α-ionylideneethane synthase for ABA biosynthesis

A critical aspect of this analysis is to reconcile our findings with the observation by Izquierdo-Bueno et al.^7^ that the deletion of *Bcstc5* abolished ABA production in an overproducing strain of *B. cinerea*. The discovery in this work of a higher-order metabolic organization provides a compelling, noncontradictory explanation. Our proteomic analysis reveals that BcStc5 is not an isolated enzyme but a central component of a densely interconnected 11-protein metabolic supercluster, which functionally links terpenoid biosynthesis, protein prenylation, and tryptophan metabolism. The concept of superclusters, where genes from multiple pathways are intertwined and coregulated, has been established in fungi like *Aspergillus fumigatus* and can lead to complex regulatory outcomes.^17^ Therefore, it is plausible that the loss of ABA production observed upon *Bcstc5* deletion was an indirect, pleiotropic effect, caused by the destabilization of this metabolic supercluster, which in turn affected the regulation or precursor supply for the spatially distinct ABA gene cluster. This hypothesis elegantly reconciles all available data without invalidating previous experimental observations.

Beyond the fundamental biological insights, this work establishes a highly efficient and scalable platform for the production of eudesmanediol (**1**). By implementing a Design of Experiments (DoE) approach, the whole-cell biocatalysis process was systematically optimized, achieving a final titer exceeding 550 mg/L in a simple lab-scale shake flask cultivation. This level of production is highly competitive with other state-of-the-art microbial systems to produce functionalized sesquiterpenoids. The PLS regression model derived from our DoE data (R^2^ = 0.95) provides more than an optimized recipe; it offers a quantitative understanding of the system’s physiology. The coefficient plot clearly visualizes the fundamental trade-off between biomass accumulation and secondary metabolite production. The conditions that maximize product titer (low temperature, low inducer concentration) are precisely those that suppress host cell growth.^30^ This empirical validation of a core principle in bioprocess engineering provides a solid, data-driven foundation for implementing two-phase cultivation strategies. The development of this high-yield process is particularly significant given the potent bioactivity of eudesmanediol (**1**). The reported antimicrobial activity against clinically relevant pathogens like MRSA and *Candida albicans* suggests its potential as a lead compound for pharmaceutical development.^37^ Our sustainable, culture-based route provides reliable access to gram-scale quantities of this molecule, overcoming the limitations of plant extraction or complex chemical synthesis and enabling the further toxicological and pharmacological studies required for its development as a therapeutic agent.

## 4 Methods

### 4.1 General Procedures and Bioinformatics

Bioinformatic analyses, including genome mining (antiSMASH 7.0),^38^ multiple sequence alignments (MAFFT),^39^ and phylogenetic tree construction (IQ-TREE),^40^ were performed as detailed in the Supplementary Methods. The amino acid sequence of BcStc5 (UniProt: G2YT23) was used as the query seed. Conserved motifs were analyzed using MEME suite^41^ and validated against the Conserved Domain Database.^42^ Homology modeling was generated using SWISS-MODEL^43^ and conservation scores were calculated via ConSurf.^44^ Molecular docking simulations were conducted using AutoDock Vina;^45^ grid parameters, receptor preparation, and binding energy scores are described in the Supplementary Methods.

### 4.2 Strain Construction and Molecular Biology

*B. cinerea* strains and plasmids used in this study are listed in Table S9. The *Bcstc5* gene was codon-optimized, cloned into pET-28a­(+), and expressed in *E. coli* BL21­(DE3) cotransformed with pJBEI-2999 for mevalonate pathway enhancement (Table S9). Detailed cloning procedures, culture conditions, and purification protocols via IMAC and size-exclusion chromatography are provided in the Supplementary Methods. Protein purity was confirmed by SDS-PAGE.

### 4.3 Chemical Characterization and Structural Elucidation

Enzymatic products were extracted with ethyl acetate and analyzed by GC–MS. For structural elucidation, compound **1** was purified by silica gel chromatography. Full 1D and 2D NMR spectroscopic data are provided in and spectra in. X-ray diffraction data were collected at 100 K. The data have been deposited in the CCDC under accession number 2494255, and structure refinement details are available in the Supplementary Methods.

### 4.4 Bioprocess Engineering and Statistical Optimization (DoE)

Fermentation parameters were optimized using a Design of Experiments (DoE) approach employing MODDE 13.1 software. A central composite design (CCD) was used to optimize temperature and induction time. An *in situ* product recovery (ISPR) strategy with isooctane was implemented to prevent product toxicity. Statistical models, ANOVA tables, and model validation parameters (*R* ^2^, *Q* ^2^) are detailed in (Optimization data in Analytical quantification was performed by HPLC-ELSD as described in the Supplementary Methods.

## 5 Conclusion

We resolved the function of BcStc5, disproving its link to ABA and identifying it as a bifunctional synthase yielding (*4S*,*5S*,*7R*,*10S*)-4β,10α-eudesmane-5β,11-diol. BcStc5 anchors a metabolic supercluster acquired via horizontal gene transfer. Furthermore, we developed a high-titer bioprocess (>550 mg/L) to access this convergent bioactive terpene.

## Supplementary Material



## Notes

Supplementary Notes and Methods: This document contains Supplementary > Notes S1–S3 regarding evolutionary implications, supercluster organization, and mechanistic analysis; detailed Supplementary Methods for bioinformatic analysis, strain construction, and structural elucidation; and the complete legends for all Supplementary Tables and Figures (DOCX). Supplementary Tables: Tables S1–S9, including genomic loci of BcStc5 homologues, docking affinity values, protein–protein interaction scores (STRING), and the experimental design matrix for DoE studies (XLSX). Supplementary Figures: Figures S1–S12, containing conserved motif analysis, GO enrichment, GC–MS identification, the HRMS and the complete set of 1D and 2D NMR spectra (^1^H, ^13^C, COSY, HMBC, HSQC, NOESY) for compound **1** (TIFF) (PDF)

## #

Institute for Mediterranean and Subtropical Horticulture “La Mayora” (IHSM), CSIC-UMA, Campus de Teatinos, Avda. Louis Pasteur, 49, 29010 Málaga, Spain

## Abbreviations

ABA: abscisic acid
BEB: *B. cinerea* Gene Expression Browser
BGC: biosynthetic gene cluster
CCF: face-centered central composite design
COSY: correlation spectroscopy
DoE: design of experiments
FDP: farnesyl diphosphate
GC-MS: gas chromatography–mass spectrometry
HGT: horizontal gene transfer
HMBC: heteronuclear multiple bond correlation
HPLC-ELSD: high-performance liquid chromatography–evaporative light scattering detection
HRMS: high-resolution mass spectrometry
HSQC: heteronuclear single quantum coherence
IPTG: isopropyl β-D-1-thiogalactopyranoside
ISPR: in situ product recovery
MFS: major facilitator superfamily
NMR: nuclear magnetic resonance
P450: cytochrome P450 monooxygenase
PLS: partial least-squares
PPI: protein–protein interaction
TLC: thin layer chromatography
TPS: terpene synthase
