# Supplementary material for: Resolution of the ABA Biosynthesis Controversy: Discovery of a Dihydroxylating Terpene Synthase and Its Convergent Evolution
Source: ACS Chem Biol. 2026 May 26;21(6):1362–72. doi: 10.1021/acschembio.6c00103 (PMC13288456; doi:10.1021/acschembio.6c00103)

Supplementary Information

Title: Resolution of the ABA Biosynthesis Controversy: Discovery of a Dihydroxylating Terpene Synthase and its Convergent Evolution

Authors: Víctor Coca-Ruiz [1,2], Katharina Hausmann [3], Gerald Dräger [4], Henry Struwe [4], Maria Zahid [3], Josefina Aleu [1,2], Dörte Solle [3], Andreas Kirschning [4,5], Isidro G. Collado [1,2], and Sascha Beutel [3]*

Affiliations: [1] Department of Organic Chemistry, Faculty of Sciences, University of Cádiz, Puerto Real, Spain. [3] Institute of Biomolecules (INBIO), University of Cádiz, Puerto Real, Spain. [4] Institute of Technical Chemistry, Leibniz University Hannover, Hannover, Germany. [5] Institute of Organic Chemistry, Leibniz University Hannover, Hannover, Germany. [6] Uppsala Biomedical Center (BMC), Uppsala University, Sweden.

Correspondence: Sascha Beutel (beutel@iftc.uni-hannover.de)

Table of Contents

1. **Supplementary Notes**
    - Note S1: Evolutionary Implications of Patchy Phylogenetic Distribution
    - Note S2: Organization of the Metabolic Supercluster and Transcriptional Regulation
    - Note S3: Detailed Mechanistic Analysis of P450-Independent Dihydroxylation
2. **Supplementary Methods**
    - **Bioinformatic Analysis**
    - **General Reagents, Strains, and Plasmids**
    - **Plasmid and Strain Construction**
    - **Whole-Cell Biocatalysis for Eudesmanediol Production**
    - **Purification and Structural Elucidation of Eudesmanediol**
3. **Supplementary Tables Legends**
    - Legends for Tables S1-S9
4. **Supplementary Figure Legends**
    - Legends for Figures S1 to S11
5. **Supplementary Figures**

1. Supplementary Notes

Note S1: Evolutionary Implications of Patchy Phylogenetic Distribution

Phylogenetic analysis of BcStc5 reveals a "patchy" distribution pattern (Table S1 and Figure 1) inconsistent with strict vertical inheritance.[1] The gene is present in generalist *Botrytis* strains (*B. cinerea*, *B. pseudocinerea*) and in distant genera like *Fusarium* and *Penicillium*, but is absent in sister *Botrytis* species that are host specialists (e.g., *B. fabae*). This pattern strongly suggests that the BcStc5 gene cluster has been mobilized via Horizontal Gene Transfer (HGT).[1]

HGT is a key driver in the evolution of fungal virulence, allowing rapid acquisition of metabolic "toolkits." In this case, syntenic conservation of the TPS/P450 gene pair across vast evolutionary distances indicates they function as an indivisible module. Selective pressure to maintain this module appears linked to the polyphagous lifestyle. *B. cinerea*, as a broad-range necrotrophic pathogen, faces massive microbial diversity in the phyllosphere and rhizosphere of its >1400 hosts. Production of eudesmanediol, a compound with broad-spectrum antimicrobial activity, would provide a direct competitive advantage, acting as a defensive antibiotic to secure the ecological niche.

Even more surprising is the **evolutionary convergence** observed with plants and bacteria. The same stereoisomer (1) is produced by the plant *Cymbopogon distans*[2] and the bacterium *Streptomyces* sp.[3,4] Since plants, bacteria, and fungi utilize distinct enzymatic machineries and precursor pathways (MEP vs. MVA), the independent evolution of the capacity to synthesize exactly the same complex molecule suggests this sesquiterpenoid possesses highly valuable intrinsic biological properties. This convergence reinforces the hypothesis that eudesmanediol is not "junk" secondary metabolite or a byproduct, but a bioactive molecule functionally optimized by natural selection across multiple kingdoms of life.[5]

Note S2: Detailed Mechanistic Analysis of P450-Independent Dihydroxylation

The formation of eudesmanediol (1) by BcStc5 represents a notable expansion of the known catalytic repertoire of fungal sesquiterpene cyclases. The central dogma of terpene biosynthesis establishes a division of labor: the terpene synthase (TPS) builds the hydrocarbon skeleton (or monohydroxylated via water quenching at the cascade's end), and subsequent oxygenases, typically cytochrome P450s, decorate this skeleton with additional functional groups. The ability of BcStc5 to install two hydroxyl groups at specific positions (C5 and C11) in a single catalytic event and without redox cofactors is exceptional.

Based on the product structure, active site modeling (Figure 2 in main text), and comparisons with the maize enzyme ZmEDS (a functionally analogous but evolutionarily distant diol synthase),[6,7] we propose a highly orchestrated sequential mechanism:

1. **Ionization and Initial Cyclization (1,10):** The reaction begins with Mg^{2+}-dependent ionization of FDP, generating the allylic farnesyl cation. Attack of the C10-C11 double bond on C1 generates the macrocyclic (E,E)-germacrenyl cation.
2. **Water Capture 1 (Hedycaryol Formation):** Instead of direct deprotonation to form germacrene A, the germacrenyl cation is trapped by a water molecule at C11. This step is critical and must be strictly controlled to prevent premature final product formation. The resulting intermediate is **(R)-hedycaryol**, a neutral alcohol.
3. **Protonation and Recycling:** To continue the reaction, neutral hedycaryol must be reactivated. We propose that conserved acidic residues in the active site, specifically the Asp301/Glu308 dyad (numbering per alignment), act as general acids to protonate the C4-C5 double bond or the C11 hydroxyl group, regenerating a reactive cation. This "stop-and-restart" strategy via a neutral intermediate is rare but not unprecedented (observed in geosmin synthesis).[8]
4. **Transannular Cyclization and Water Capture 2:** The reactivated intermediate undergoes a second cyclization (eudesmane) followed by a final water capture at C5. The specific stereochemistry of the product (4S,5S,7R,10S) dictates that water must attack from the *beta* face, an orientation facilitated by the hydrophobic pocket architecture restricting solvent access to a single trajectory.

ConSurf analysis (Supplementary Figure S3) reveals extreme evolutionary conservation not only in metal-binding motifs but also in aromatic residues (Tyr, Phe, Trp) lining the catalytic cavity. These residues likely stabilize multiple carbocation intermediates via cation-π interactions, preventing unwanted side reactions.[9,10] The absence of a P450 in the *in vitro* reaction confirms that all this chemical complexity is encoded within the unique structure of BcStc5.

Note S3: Organization of the Metabolic Supercluster and Transcriptional Regulation

The genomic context of *Bcstc5* reveals a previously unappreciated layer of metabolic integration in *B. cinerea*.[11] The gene is part of a "metabolic supercluster" that physically and functionally links three traditionally distinct biosynthetic pathways: (1) sesquiterpenoid biosynthesis (centered on *Bcstc5*), (2) protein prenylation machinery, and (3) tryptophan biosynthesis.[11]

Protein-protein interaction (PPI) network analysis (see Figure 3) demonstrates that these proteins form a high-confidence interactome. We hypothesize that this organization facilitates "substrate channeling" or coordinated regulation.[11] The inclusion of tryptophan biosynthetic genes (e.g., anthranilate synthase) within this cluster is particularly intriguing, as tryptophan is a precursor to indole-derived secondary metabolites and several plant growth regulators.

Transcriptional data suggests that this supercluster is regulated as a single unit, likely under the control of specific transcription factors sensitive to the host environment. This integration ensures that when the fungus initiates the production of eudesmanediol, it simultaneously optimizes the supply of isoprenoid precursors (via the prenylation module) and coordinates this with its wider primary and secondary metabolism. This "supercluster" architecture represents a sophisticated evolutionary strategy to ensure metabolic efficiency during host invasion.

2. Supplementary Methods

2.1. Bioinformatic Analysis

2.1.1. Biosynthetic Gene Cluster (BGC) Identification and Annotation

A genome mining survey was conducted to identify conserved BGCs across a curated set of publicly available fungal genomes. This set included strains of *Botrytis cinerea* (B05.10, T4, DW1), other sequenced species of the *Botrytis* genus, and representative species from other genera, including *Sclerotinia*, *Fusarium*, *Penicillium*, and *Thelonectria*. Complete genomes were analyzed using the antiSMASH platform (fungal version 7.0)[12] to predict BGC boundaries and annotate gene functions. The resulting BGC predictions were manually inspected to identify clusters containing a syntenically conserved gene pair encoding a putative terpene synthase (TS) and a cytochrome P450 (P450).

2.1.2. Homolog Identification and Phylogenetic Analysis

Following the identification of the conserved BGC, the terpene synthase from *B. cinerea* B05.10, BcStc5 (UniProt: G2YT23), was selected for detailed evolutionary analysis. Orthologs were identified via a BLASTp search against the NCBI non-redundant (nr) protein database,[13,14] using stringency criteria of sequence identity > 30% and query coverage > 70%.[15] A dataset of 274 orthologous sequences was compiled and aligned using MAFFT (v7.450) with the L-INS-i algorithm.[16] A maximum likelihood phylogenetic tree was constructed from the alignment using IQ-TREE (v2.0.3).[17] The best-fit amino acid substitution model was selected by the integrated ModelFinder tool based on the Bayesian Information Criterion (BIC).[18] Branch support was assessed with 1,000 ultrafast bootstrap (UFBoot2) replicates.[19] The final annotated tree was visualized using the Interactive Tree Of Life (iTOL) v6 web server.[20]

2.1.3. Sequence Motif and Structural Conservation Analysis

Conserved sequence motifs within the 274-ortholog dataset were identified using the MEME suite (v5.1.1).[21] The functional identities of the resulting motifs were validated by comparison against the JASPAR/UniProt database using the TOMTOM algorithm,[22] confirming the presence of the canonical Class I terpene cyclase motifs DDxxD and (N,D)Dxx(S,T)xxxE. To map evolutionary pressures onto the protein structure, sequence conservation was calculated and projected onto a homology model of BcStc5 using the ConSurf web server.[23] The analysis used a pre-calculated multiple sequence alignment from the UniRef database.[24]

2.1.4. Functional Context and Network Analysis

To investigate the functional context of BcStc5, protein-protein interaction (PPI) networks were predicted for *both B. cinerea* strain T4 and the reference strain DW1 using the STRING database (v11.5).[25] The query sets consisted of the eleven selected biosynthetic enzymes from *B. cinerea* T4 and their respective orthologs in *B. cinerea* DW1. Only high-confidence interactions (score > 0.800) were retained for analysis. The cohesion of these predicted superclusters was computationally validated using the Markov Cluster Algorithm (MCL, v14-137) with an inflation parameter of 2.0. Functional enrichment for each network was analyzed using the integrated Gene Ontology (GO) tool.[26] GO terms for "Biological Process" were considered significantly enriched if the False Discovery Rate (FDR) was less than 1.0×10^{-9}.

For comparative transcriptomics, the expression profile of *BcStc5* was compared to that of *BcBot2*, the core sesquiterpene cyclase responsible for botrydial biosynthesis, using curated RNA-Seq data from the *Botrytis cinerea* Gene Expression Browser (BEB).[27]

2.1.5. Molecular Docking

Docking simulations were performed using AutoDock Vina (version 1.1.2),[28] which applies a gradient-based search algorithm coupled with a scoring function to estimate ligand binding affinities (kcal/mol). An exhaustiveness value of 10 was used to balance sampling thoroughness with computational efficiency.[29] The three-dimensional (3D) structure of BcStc5 (UniProt ID: A0A384J4Z3) was retrieved from the Protein Data Bank (PDB).[30] Prior to docking, non-essential components—including crystallographic water molecules, heteroatoms, and co-crystallized ligands—were removed using ChimeraX (version 1.10.1).[31] Polar hydrogen atoms were added, and Kollman charges were assigned using AutoDock Tools (MGLTools, version 1.5.7).[28] The prepared protein structure was then saved in PDBQT format, as required by AutoDock Vina.[28] During docking, the protein structure was treated as rigid. Ligand structures were obtained from the PubChem database[32] in SDF format. For example, the FDP structure (CID: 445713) was converted into 3D coordinates and geometry-optimized using Avogadro. Further processing with ADT included assignment of Gasteiger partial charges, definition of rotatable bonds, and conversion into PDBQT format. A docking grid

was defined to encompass the predicted binding pocket, guided by either the location of a co-crystallized ligand or key functional residues. The grid box was centered at:

- center_x = 0.0, center_y = 0.0, center_z = 0.0
  with dimensions:
- size_x = 40 Å, size_y = 25 Å, size_z = 25 Å.

These parameters were specified in a configuration file for AutoDock Vina. Binding affinities were estimated using Vina's scoring function. The top-ranked poses were selected for further analysis and evaluation of docking reliability, including RMSD calculations where applicable.

2.2. General Reagents, Strains, and Plasmids

All chemical products, solvents, and reagents were procured in analytical grade or higher from either Sigma-Aldrich (Merck) or Carl Roth, unless otherwise specified. Solvents for High-Performance Liquid Chromatography (HPLC) and Nuclear Magnetic Resonance (NMR) were of the respective specialized grades. The genomic sequence for the target gene, BcStc5, was obtained from the reference genome of *B. cinerea* strain B05.10 (Assembly GCA_000143535.4). Standard molecular biology procedures were performed using *E. coli* DH5α as the cloning host and *E. coli* BL21(DE3) as the expression host. All bacterial strains and plasmids central to this work are enumerated in Table 5.

2.3. Plasmid and Strain Construction

The coding sequence for *BcStc5* from *B. cinerea* B05.10 was synthesized by BioCat GmbH with codon usage optimized for expression in *E. coli*. The synthetic gene was cloned into the pET-28a(+) expression vector using NdeI and XhoI restriction sites, yielding the plasmid pET-28a-*BcStc5*. This construct encodes an N-terminally His_6-tagged BcStc5 protein under the control of an IPTG-inducible T7 promoter. The plasmid was propagated in *E. coli* DH5α and its sequence integrity was confirmed by Sanger sequencing.

For whole-cell biocatalysis, a metabolically engineered production host was generated. *E. coli* BL21(DE3) was co-transformed by electroporation with the expression plasmid pET-28a-*BcStc5* (KanR) and the precursor-supplying plasmid pJBEI-2999 (CmR). The pJBEI-2999 plasmid encodes the heterologous mevalonate (MVA) pathway, ensuring a robust supply of the farnesyl diphosphate (FDP) precursor that circumvents potential flux limitations of the native *E. coli* MEP pathway. Co-transformants were selected and maintained on Luria-Bertani (LB) agar containing kanamycin (50 µg/mL) and chloramphenicol (34 µg/mL).

2.4. Whole-Cell Biocatalysis for Eudesmanediol Production

The production and recovery of sesquiterpenes were performed by adapting the protocol established by

Wildhagen *et al.* (2023)[33] with modifications. Briefly, a single colony of the engineered *E. coli* BL21(DE3) strain was used to inoculate a 5 mL LB pre-culture containing appropriate antibiotics, which was incubated overnight at 37 °C. This was subsequently used to inoculate production cultures in an optimized culture medium (TB medium). Cultures were grown at 37 °C with agitation until reaching an optical density at 600 nm (OD_{600}) of 0.6–0.8 rel. AU.

Protein expression was induced by the addition of isopropyl-β-D-1-thiogalactopyranoside (IPTG), and the cultivation temperature was simultaneously reduced from 30ºC to 16°C to enhance protein folding and solubility. For *in situ* product recovery (ISPR), an organic overlay of isooctane (10% v/v) was added at the time of induction to sequester the hydrophobic sesquiterpene product. The culture was continued for an additional 24–72 hours.

2.5. Purification and Structural Elucidation of Eudesmanediol

2.5.1. Isolation and Purification

Following cultivation, the isooctane layer was separated by centrifugation and dried over anhydrous Na_2SO_4, filtered, and concentrated under reduced pressure. The crude extract was purified by silica gel column chromatography (Silica Gel 60, 230–400 mesh) using a gradient elution of pentane and diethyl ether. Fractions containing the target compound were pooled and concentrated to yield eudesmanediol (**1**) as a white crystalline solid.

2.5.2 Spectroscopic and Spectrometric Analysis

Gas chromatography-mass spectrometry (GC-MS) analyses were performed on an Agilent 7890B GC system coupled to a 5977B MSD, equipped with an HP-5ms capillary column (30 m x 0.25 mm, 0.25 µm film thickness). The temperature program was: 50 °C (1 min), ramp at 20 °C/min to 300 °C (6 min). Analysis for high-resolution mass spectrometry (HR-ESI-MS) were conducted on an UHPLC separation system ACQUITY UPLC H-Class system, with a binary solvent system and an automatic sample manager equipped with a UPLC BEH C18 (2.1 mm × 100 mm, 1.7 mm) column, at a temperature of 55 ◦C. The mobile phases were obtained by mixing eluent A (water) and eluent B (0.1% formic acid in acetonitrile, v/v). These phases were delivered at a flow rate of 0.4 mL/min by means of a mixing linear gradient program as follows: 0–5 min, 80-0% A; 5–8.5 min, 0% A; 8.5–8 min, 0-80% A. The injection volume of all samples was 2 µL.

UHPLC system was linked to a quadrupole time-of-flight tandem high-resolution mass spectrometer (Synapt-XS QTOF; Waters, Manchester, UK) equipped with an ESI source, in positive mode. ESI parameters: sample cone voltage of 30 V; source temperature of 120 ◦C; cone gas flow of 50 L/h; desolvation gas flow of 850 L/h; capillary voltage 0.7 kV and desolvation temperature was set at 450 ºC.

Mass accuracy and reproducibility were achieved by mass spectrometer calibration over a 100–1200Da mass range, using sodium formiate solution. Leucine-enkephalin (m/z 556.2771 in positive-ion mode) was used as the external reference of LockSpray, infused at a constant flow of 10 µL/min. Calibration was made considering ions as atom aggregates.

Data obtained from UHPLC/HR-ESI-MS experiments were used to determine the exact molecular formula for compound of interest.[34] An ion corresponding to the sodium adduct, $[M+Na]^+$, was observed at m/z 263.1986. This experimental mass is consistent with the calculated mass of 263.1987 for $C_{15}H_{28}O_2Na$, thereby confirming the molecular formula of the neutral compound as $C_{15}H_{28}O_2$.

NMR spectra (^1H, ^{13}C, COSY, HMBC, HSQC and NOESY) were recorded on a Bruker Avance III spectrometer (600 MHz for ^1H, 151 MHz for ^{13}C) in deuterated benzene (C_6D_6). Chemical shifts (δ) are reported in ppm relative to the residual solvent signal (δH 7.16, δC 128.06).

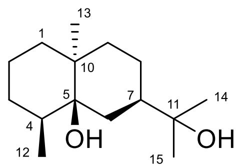

1

$C_{15}H_{28}O_2$

240.39

(4*S*,5*S*,7*R*,10*S*)-4β,10α-Eudesmane-5β,11-diol (1): White crystals; m.p. 123 °C; $[\alpha]_D^{26}$ -10.6 (*c* 0.31, $CHCl_3$, reported -9.0, *c* 0.8, $CHCl_3$); IR (film) vmax 3299, 2977, 2937, 2861, 1632, 1447, 1376 cm^{-1}; ^1H NMR (600 MHz, C_6D_6) δ = 4.24 (1H, s_{br}, H_{OH}), 4.06 (1H, s_{br}, H_{OH}), 2.05 (1H, td, *J* = 13.6 Hz, 4.7 Hz, H_9), 2.00 (1H, d_{br}, *J* = 15.0 Hz, H_6), 1.92 – 1.87 (1H, m, H_8), 1.76 (1H, td, *J* = 13.2, 4.3 Hz, H_1), 1.70 – 1.63 (1H, m, H_8), 1.63 – 1.56 (1H, m, H_2), 1.58 – 1.53 (1H, m, H_4), 1.52 – 1.49 (1H, m, H_3), 1.47 – 1.43 (1H, m, H_2), 1.41 (1H, t, J = 7.8 Hz, H_7), 1.37 – 1.33 (1H, m, H_3), 1.29 (1H, dd, *J* = 15.0, 7.9 Hz, H_6), 1.26 (3H, s, H_{14} or H_{15}), 1.23 (3H, s, H_{14} or H_{15}), 0.95 (3H, s, H_{13}), 0.94 – 0.92 (2H, m, H_1, H_9), 0.92 (3H, d, *J* = 6.2 Hz, H_{12}) ppm; ^{13}C NMR (151 MHz, C_6D_6) δ = 73.6 (C_5), 73.3 (C_{11}), 41.3 (C_7), 37.3 (C_{10}), 35.1 (C_1), 34.7 (C_4), 34.6 (C_9), 30.9 (C_3), 30.2 (C_{14} or C_{15}), 30.0 (C_6), 29.8 (C_{14} or C_{15}), 21.3 (C_2), 20.9 (C_{13}), 20.6 (C_8), 15.3 (C_{12}) ppm; HRMS (ESI-TOF) m/z: $[M+Na]^+$ calc. for $C_{15}H_{28}O_2Na$: 263.1987; found: 263.1986.

2.5.3. Single-Crystal X-ray analysis

Single colorless needle-shaped crystals of 1 were crystallized by slow evaporation from ethyl acetate / n-hexane. A suitable crystal 0.70×0.11×0.08 mm^3 was selected and mounted on a 18 mm mounted CryoLoop (20 micron, 0.2 - 0.3 mm, Hampton Research) on an XtaLAB AFC12 (RINC): Kappa single diffractometer. The crystal was kept at a steady T = 99.99(10) K during data collection. The structure was solved with the ShelXT[35] structure solution program using the Intrinsic Phasing solution method and by using Olex2[36] as

the graphical interface. The model was refined with version 2019/3 of ShelXL 2019/3[37] using Least Squares minimization.

Crystal Data. $C_{15}H_{28}O_2$, Mr = 240.37, trigonal, $P3_221$ (No. 154), a = 10.9746(2) Å, b = 10.9746(2) Å, c = 20.3645(5) Å, α = 90 °, β = 90 °, γ = 120°, V = 2124.13(9) Å^3, T = 99.99(10) K, Z = 6, Z' = 1, μ(Cu Kα) = 0.558, 33010 reflections measured, 3047 unique (Rint = 0.0814) which were used in all calculations. The final wR_2 was 0.0907 (all data) and R_1 was 0.0332 (I > 2(I)).

2.5.4. Bioprocess Optimization using Design of Experiments (DoE)

The optimization of eudesmanediol (**1**) production was conducted using a statistical Design of Experiments (DoE) approach.

First, a screening of variables was performed using a 2^3 fractional factorial design with three central replicates to evaluate the influence of post-induction time (t), cold-shift induction temperature (ΔT), IPTG concentration ([IPTG]), and initial glycerol concentration ([GC]).

As IPTG and glycerol showed no significant effects, a second optimization phase was implemented using a Face-Centered Central Composite Design (CCF). This design, consisting of 11 experiments with three central replicates, was used to model the influence of the two most significant process variables: post-induction cultivation time (t, ranging from 2–4 days) and induction temperature (ΔT, corresponding to final temperatures of 12°C to 20°C). All experiments were performed in 100 mL shake flasks containing 25 mL of culture medium. Product quantification was performed by GC-FID, and the data were analyzed by PLS regression using MODDE 13.1 software (Sartorius). The complete experimental matrix is detailed in Supplementary Table S6.

2.5.5. *In Vitro* Enzymatic Assay

The production and recovery of sesquiterpenes were performed by adapting the protocol established by Moeller *et al.* (2024) and Struwe *et al.* (2025)[38,39] with modifications. Briefly, *E. coli* BL21(DE3) cells harboring only pET-28a-*BcStc5* were cultured and induced. Cells were harvested, resuspended in lysis buffer (50 mM Tris-HCl pH 8.0, 300 mM NaCl, 10 mM imidazole), and lysed by sonication. The soluble His_6-BcStc5 protein was purified from the cell-free lysate using an Äkta pure system. The purification was performed via immobilized metal affinity chromatography (IMAC) using a His-Trap FF column (Cytiva). The protein was eluted with an imidazole gradient, followed by buffer exchange into storage buffer (50 mM Tris-HCl pH 8.0, 300 mM NaCl, 10% glycerol) using a desalting column.

Enzymatic assays were performed in 500 µL glass vials containing assay buffer (50 mM HEPES, pH 7.5, 10 mM $MgCl^2$, 10% glycerol), 150 µM of (*E*,*E*)-Farnesyl diphosphate (FDP), and 0.1 mg/mL of purified BcStc5. Reactions were incubated at 37 °C for 30–60 minutes, quenched with 2 M NaCl, and extracted with ethyl acetate. The organic phase was dried over Na_2SO_4, concentrated, and analyzed by GC-MS.

2.5.6. Data Availability

The crystallographic data for compound **1** have been deposited in the Cambridge Crystallographic Data Centre (CCDC) under deposition number CCDC 2494255. All other data supporting the findings of this study are available within the article and its Supplementary Information files or from the corresponding author upon reasonable request.

References

(1) Dhillon, B.; Feau, N.; Aerts, A. L.; Beauseigle, S.; Bernier, L.; Copeland, A.; Foster, A.; Gill, N.; Henrissat, B.; Herath, P.; LaButti, K. M.; Levasseur, A.; Lindquist, E. A.; Majoor, E.; Ohm, R. A.; Pangilinan, J. L.; Pribowo, A.; Saddler, J. N.; Sakalidis, M. L.; de Vries, R. P.; Grigoriev, I. V.; Goodwin, S. B.; Tanguay, P.; Hamelin, R. C. Horizontal gene transfer and gene dosage drives adaptation to wood colonization in a tree pathogen. *Proc. Natl. Acad. Sci.* **2015**, *112* (11), 3451–3456. https://doi.org/10.1073/pnas.1424293112.

(2) Mathela, C. S.; Melkani, A. B.; Pant, A.; Dev, V.; Nelson, T. E.; Hope, H.; Bottini, A. T. A eudesmanediol from *Cymbopogon distans*. *Phytochemistry* **1989**, *28* (3), 936–938. https://doi.org/10.1016/0031-9422(89)80148-7.

(3) Ding, L.; Hertweck, C. Oxygenated geosmins and plant-like eudesmanes from a bacterial mangrove endophyte. *J. Nat. Prod.* **2020**, *83* (7), 2207–2211. https://doi.org/10.1021/acs.jnatprod.0c00304.

(4) Ding, N.; Jiang, Y.; Han, L.; Chen, X.; Ma, J.; Qu, X.; Mu, Y.; Liu, J.; Li, L.; Jiang, C.; Huang, X. Bafilomycins and odoriferous sesquiterpenoids from *Streptomyces Albolongus* isolated from *Elephas Maximus* feces. *J. Nat. Prod.* **2016**, *79* (4), 799–805. https://doi.org/10.1021/acs.jnatprod.5b00827.

(5) Karunanithi, P. S.; Zerbe, P. Terpene synthases as metabolic gatekeepers in the evolution of plant terpenoid chemical diversity. *Front. Plant Sci.* **2019**, *10*, 1166. https://doi.org/10.3389/fpls.2019.01166.

(6) Liang, J.; Liu, J.; Brown, R.; Jia, M.; Zhou, K.; Peters, R. J.; Wang, Q. Direct production of dihydroxylated sesquiterpenoids by a maize terpene synthase. *Plant J.* **2018**, *94* (5), 847–856. https://doi.org/10.1111/tpj.13901.

(7) Liang, J.; Wang, L.; Liu, J.; Shen, Q.; Fu, J.; Peters, R. J.; Wang, Q. Probing enzymatic structure and function in the dihydroxylating sesquiterpene synthase ZmEDS. *Biochemistry* **2020**, *59* (28), 2660–2666. https://doi.org/10.1021/acs.biochem.0c00395.

(8) Ding, N.; Han, L.; Jiang, Y.; Li, G.; Liu, J.; Mu, Y.; Huang, X. Sesquiterpenoids from S*treptomyces anulatus* isolated from *Giraffa camelopardalis* feces. *Magn. Reson. Chem.* **2018**, *56* (5), 352–359.

https://doi.org/10.1002/mrc.4709.

(9) Christianson, D. W. Structural and chemical biology of terpenoid cyclases. *Chem. Rev.* **2017**, *117* (17), 11570–11648. https://doi.org/10.1021/acs.chemrev.7b00287.

(10) Christianson, D. W. Structural biology and chemistry of the terpenoid cyclases. *Chem. Rev.* **2006**, *106* (8), 3412–3442. https://doi.org/10.1021/cr050286w.

(11) Wiemann, P.; Guo, C.-J.; Palmer, J. M.; Sekonyela, R.; Wang, C. C. C.; Keller, N. P. Prototype of an intertwined secondary-metabolite supercluster. *Proc. Natl. Acad. Sci. U. S. A.* **2013**, *110* (42), 17065–17070. https://doi.org/10.1073/pnas.1313258110.

(12) Blin, K.; Shaw, S.; Augustijn, H. E.; Reitz, Z. L.; Biermann, F.; Alanjary, M.; Fetter, A.; Terlouw, B. R.; Metcalf, W. W.; Helfrich, E. J. N.; van Wezel, G. P.; Medema, M. H.; Weber, T. AntiSMASH 7.0: New and improved predictions for detection, regulation, chemical structures and visualisation. *Nucleic Acids Res.* **2023**, *51* (W1), W46–W50. https://doi.org/10.1093/nar/gkad344.

(13) Altschul, S. Gapped BLAST and PSI-BLAST: A new generation of protein database search programs. *Nucleic Acids Res.* **1997**, *25* (17), 3389–3402. https://doi.org/10.1093/nar/25.17.3389.

(14) Camacho, C.; Coulouris, G.; Avagyan, V.; Ma, N.; Papadopoulos, J.; Bealer, K.; Madden, T. L. BLAST+: Architecture and applications. *BMC Bioinformatics* **2009**, *10* (1), 421. https://doi.org/10.1186/1471-2105-10-421.

(15) Pearson, W. R. An Introduction to sequence similarity (“homology”) searching. *Curr. Protoc. Bioinforma.* **2013**, *42* (1). https://doi.org/10.1002/0471250953.bi0301s42.

(16) Katoh, K.; Standley, D. M. MAFFT Multiple Sequence Alignment Software Version 7: improvements in performance and usability. *Mol. Biol. Evol.* **2013**, *30* (4), 772–780. https://doi.org/10.1093/molbev/mst010.

(17) Wong, T.; Ly-Trong, N.; Ren, H.; Baños, H.; Roger, A.; Susko, E.; Bielow, C.; De Maio, N.; Goldman, N.; Hahn, M.; Huttley, G.; Lanfear, R.; Minh, B. Q. IQ-TREE 3: Phylogenomic inference software using complex evolutionary models. April 7, 2025. https://doi.org/10.32942/X2P62N.

(18) Kalyaanamoorthy, S.; Minh, B. Q.; Wong, T. K. F.; von Haeseler, A.; Jermiin, L. S. ModelFinder: Fast model selection for accurate phylogenetic estimates. *Nat. Methods* **2017**, *14* (6), 587–589. https://doi.org/10.1038/nmeth.4285.

(19) Hoang, D. T.; Chernomor, O.; von Haeseler, A.; Minh, B. Q.; Vinh, L. S. UFBoot2: Improving the ultrafast bootstrap approximation. *Mol. Biol. Evol.* **2018**, *35* (2), 518–522. https://doi.org/10.1093/molbev/msx281.

(20) Letunic, I.; Bork, P. Interactive Tree of Life (ITOL) v6: Recent updates to the phylogenetic tree display and annotation tool. *Nucleic Acids Res.* **2024**, *52* (W1), W78–W82. https://doi.org/10.1093/nar/gkae268.

(21) Bailey, T. L.; Elkan, C. Fitting a mixture model by expectation maximization to discover motifs in biopolymers. *Proceedings. Int. Conf. Intell. Syst. Mol. Biol.* **1994**, *2*, 28–36.

(22) Gupta, S.; Stamatoyannopoulos, J. A.; Bailey, T. L.; Noble, W. S. Quantifying similarity between motifs. *Genome Biol.* **2007**, *8* (2), R24. https://doi.org/10.1186/gb-2007-8-2-r24.

(23) Yariv, B.; Yariv, E.; Kessel, A.; Masrati, G.; Chorin, A. Ben; Martz, E.; Mayrose, I.; Pupko, T.; Ben-Tal, N. Using evolutionary data to make sense of macromolecules with a “face-lifted” ConSurf. *Protein Sci.* **2023**, *32* (3), e4582. https://doi.org/10.1002/pro.4582.

(24) Suzek, B. E.; Wang, Y.; Huang, H.; McGarvey, P. B.; Wu, C. H. UniRef Clusters: A comprehensive and scalable alternative for improving sequence similarity searches. *Bioinformatics* **2015**, *31* (6), 926–932. https://doi.org/10.1093/bioinformatics/btu739.

(25) Szklarczyk, D.; Kirsch, R.; Koutrouli, M.; Nastou, K.; Mehryary, F.; Hachilif, R.; Gable, A. L.; Fang, T.; Doncheva, N. T.; Pyysalo, S.; Bork, P.; Jensen, L. J.; von Mering, C. The string database in 2023: Protein–protein association networks and functional enrichment analyses for any sequenced genome of interest. *Nucleic Acids Res.* **2023**, *51* (D1), D638–D646. https://doi.org/10.1093/nar/gkac1000.

(26) Thomas, P. D.; Ebert, D.; Muruganujan, A.; Mushayahama, T.; Albou, L.; Mi, H. PANTHER: Making genome-scale phylogenetics accessible to all. *Protein Sci.* **2022**, *31* (1), 8–22. https://doi.org/10.1002/pro.4218.

(27) Pérez-Lara, G.; Moyano, T. C.; Vega, A.; Larrondo, L. F.; Polanco, R.; Álvarez, J. M.; Aguayo, D.; Canessa, P. The *Botrytis cinerea* gene expression browser. *J. Fungi* **2023**, *9* (1), 84. https://doi.org/10.3390/jof9010084.

(28) Trott, O.; Olson, A. J. AutoDock Vina: Improving the speed and accuracy of docking with a new scoring function, efficient optimization, and multithreading. *J. Comput. Chem.* **2010**, *31* (2), 455–461. https://doi.org/10.1002/jcc.21334.

(29) Bugnon, M.; Röhrig, U. F.; Goullieux, M.; Perez, M. A. S.; Daina, A.; Michielin, O.; Zoete, V. SwissDock 2024: Major enhancements for small-molecule docking with attracting cavities and AutoDock Vina. *Nucleic Acids Res.* **2024**, *52* (W1), W324–W332. https://doi.org/10.1093/nar/gkae300.

(30) Fermi, G.; Perutz, M. F. The crystal structure of human deoxyhaemoglobin at 1.74 angstroms resolutioN. *Worldwide Protein Data Bank*. July 17, 1984. https://doi.org/10.2210/pdb4hhb/pdb.

(31) Pettersen, E. F.; Goddard, T. D.; Huang, C. C.; Meng, E. C.; Couch, G. S.; Croll, T. I.; Morris, J. H.; Ferrin, T. E. UCSF ChimeraX: Structure visualization for researchers, educators, and developers. *Protein Sci.* **2021**, *30* (1), 70–82. https://doi.org/10.1002/pro.3943.

(32) Kim, S.; Chen, J.; Cheng, T.; Gindulyte, A.; He, J.; He, S.; Li, Q.; Shoemaker, B. A.; Thiessen, P.

A.; Yu, B.; Zaslavsky, L.; Zhang, J.; Bolton, E. E. PubChem 2025 update. *Nucleic Acids Res.* **2025**, *53* (D1), D1516–D1525. https://doi.org/10.1093/nar/gkae1059.

(33) Wildhagen, M.; Pudenz, T.; Nguyen, T.; Kirschning, A.; Beutel, S. Biokatalytische ganzzellproduktion des sesquiterpens presilphiperfolan-8β-ol in stoffwechseloptimierten *Escherichia coli*. *Chemie Ing. Tech.* **2023**, *95* (4), 576–586. https://doi.org/10.1002/cite.202200115.

(34) *How to use the Elemental Composition application in MassLynx - WKB17472 - Waters*. https://support.waters.com/KB_Inf/MassLynx/WKB17472_How_to_use_Elemental_Composition_application_in_MassLynx (accessed 2025-10-27).

(35) Sheldrick, G. M. Crystal structure refinement with SHELXL. *Acta Crystallogr. Sect. C Struct. Chem.* **2015**, *71* (1), 3–8. https://doi.org/10.1107/S2053229614024218.

(36) Dolomanov, O. V.; Bourhis, L. J.; Gildea, R. J.; Howard, J. A. K.; Puschmann, H. OLEX2 : A complete structure solution, refinement and analysis program. *J. Appl. Crystallogr.* **2009**, *42* (2), 339–341. https://doi.org/10.1107/S0021889808042726.

(37) Lübben, J.; Wandtke, C. M.; Hübschle, C. B.; Ruf, M.; Sheldrick, G. M.; Dittrich, B. Aspherical Scattering Factors for SHELXL – Model, implementation and application. *Acta Crystallogr. Sect. A Found. Adv.* **2019**, *75* (1), 50–62. https://doi.org/10.1107/S2053273318013840.

(38) Moeller, M.; Dhar, D.; Dräger, G.; Özbasi, M.; Struwe, H.; Wildhagen, M.; Davari, M. D.; Beutel, S.; Kirschning, A. Sesquiterpene cyclase BcBOT2 promotes the unprecedented wagner-meerwein rearrangement of the methoxy group. *J. Am. Chem. Soc.* **2024**, *146* (26), 17838–17846. https://doi.org/10.1021/jacs.4c03386.

(39) Struwe, H.; Grimm, C.; Dräger, G.; Beutel, S.; Alcalde, M.; Kirschning, A.; Kara, S. Expanding the "Terpenome": Applications of unspecific peroxygenases (UPOs) in oxidations of unnatural terpenoids. *ChemCatChem* **2025**, *17* (1). https://doi.org/10.1002/cctc.202401414.

3. Supplementary Tables

Due to their large size and format, all Supplementary Tables (S1–S9) are provided as a single standalone dataset hosted on the Zenodo repository. They are available free of charge and can be accessed or downloaded via the following Digital Object Identifier (DOI):

Dataset DOI: https://doi.org/10.5281/zenodo.19614331

List of Available Tables in the Dataset:

- **Table S1:** Distribution of the BcStc family among the Botrytis genera with NCBI ID.

- **Table S2:** List of key homologs of BcStc5 identified in other fungi.
- **Table S3:** Detailed results of the ConSurf analysis for BcStc5, including conservation scores and functional/structural predictions for each residue.
- **Table S4:** Calculated binding affinity (ΔG) and root mean square deviation (RMSD) values for the top five docking models of FDP in the BcStc5 active site.
- **Table S5:** Complete list of protein-protein interactions and associated STRING scores for the supercluster networks: (A) Dw1 and (B) T4.
- **Table S6:** Functional annotation and enrichment analysis (GO terms, KEGG pathways, and STRING clusters) for the supercluster proteins: (A) *B. cinerea* DW1 and (B) *B. cinerea* T4.
- **Table S7:** Functional Conservation of the Metabolic Supercluster in *B. cinerea* Strains T4 and DW1.
- **Table S8:** Experimental design matrix and observed responses for the DoE (Design of Experiments) study.
- **Table S9:** Bacterial Strains and Plasmids Utilized in This Study.

4. Supplementary Figure Legends

Supplementary Figure S1. Experimental matrix for the comparative transcriptomic analysis of *Bcstc5* and *Bcbot2*.

Supplementary Figure S2. (**A**) MEME logo of the conserved motif of BcStc5. (**B**) Conserved motifs in the terpene synthase (BcStc5) orthologs identified by MEME analysis.

Supplementary Figure S3. ConSurf analysis of BcStc5 showing evolutionary conservation of amino acid residues.

Supplementary Figure S4. Gene Ontology (GO) enrichment analysis for Biological Process terms associated with the 11-protein supercluster for *B. cinerea* (**A**) Dw1 and (**B**) T4.

Supplementary Figure S5. Identification of the BcStc5 product as a sesquiterpenoid diol by gas chromatography-mass spectrometry (GC-MS).

Supplementary Figure S6. High-resolution mass spectrum (HR-ESI-MS) of eudesmanediol (1). The spectrum displays the sodium adduct $[M+Na]^+$ at *m/z* 263.1986, consistent with the calculated exact mass for the molecular formula C15H28O2Na (263.1987).

Supplementary Figure S7. ^{1}H NMR spectrum (600 MHz) of **1** in benzene-d_6 at 298 K.

Supplementary Figure S8. Proton-decoupled ^{13}C ^{1}H NMR spectrum (151 MHz) of **1** in benzene-d_6 at 298 K.

Supplementary Figure S9. ^{1}H-^{1}H COSY spectrum of **1** in benzene-d_6 (600 MHz, 298 K).

Supplementary Figure S10. HMBC spectrum of **1** in benzene-d_6 (600 MHz, 298 K).

Supplementary Figure S11. Multiplicity-edited HSQC spectrum of **1** in benzene-d_6 (600 MHz, 298 K). Positive correlations for CH/CH_3 groups are shown in black, while negative correlations for CH_2 groups are shown in grey.

Supplementary Figure S12. ^{1}H-^{1}H NOESY spectrum of 1 in benzene-d_6 (600 MHz, 298 K).

5. Supplementary Figures

Figure S1.

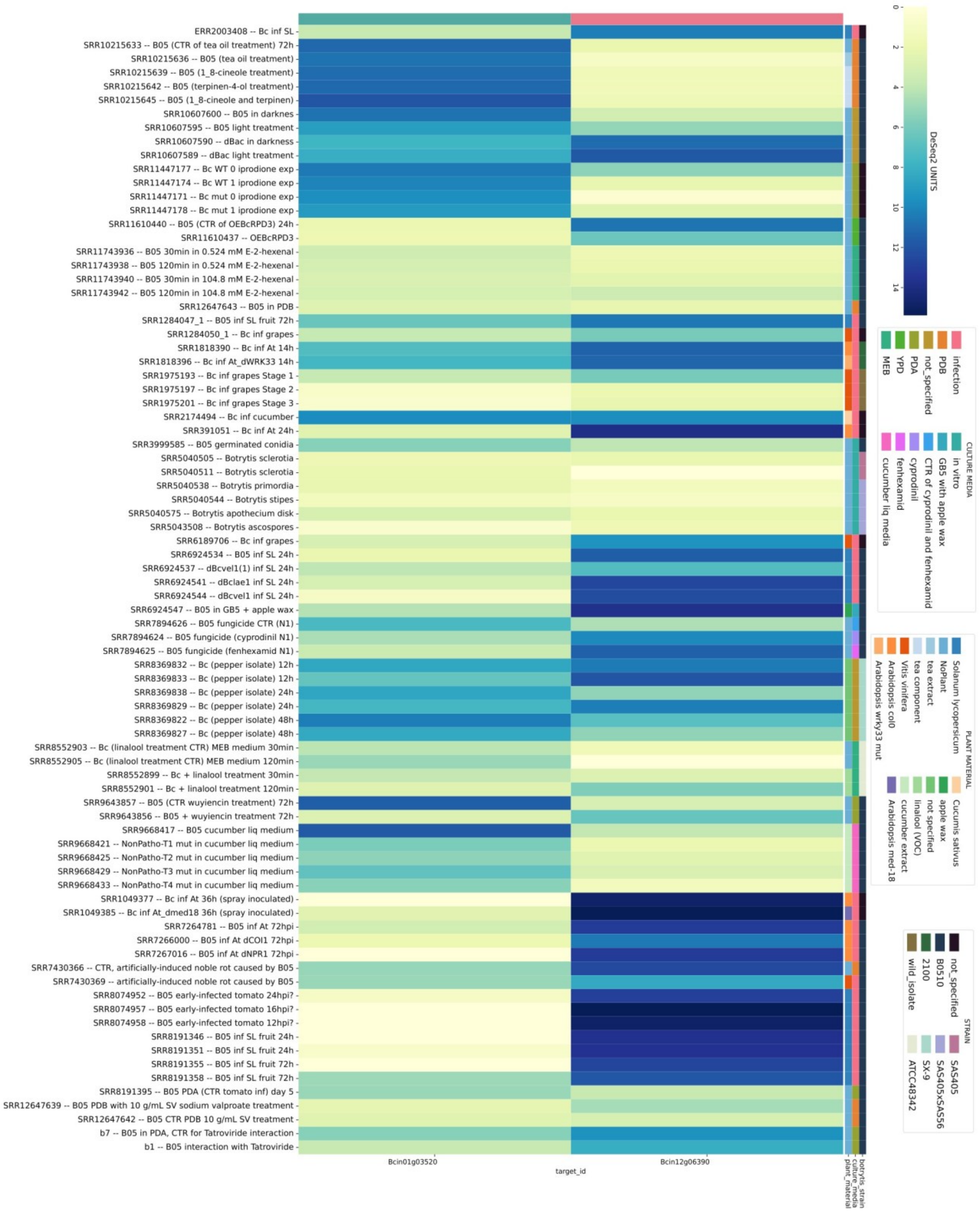

Figure S2

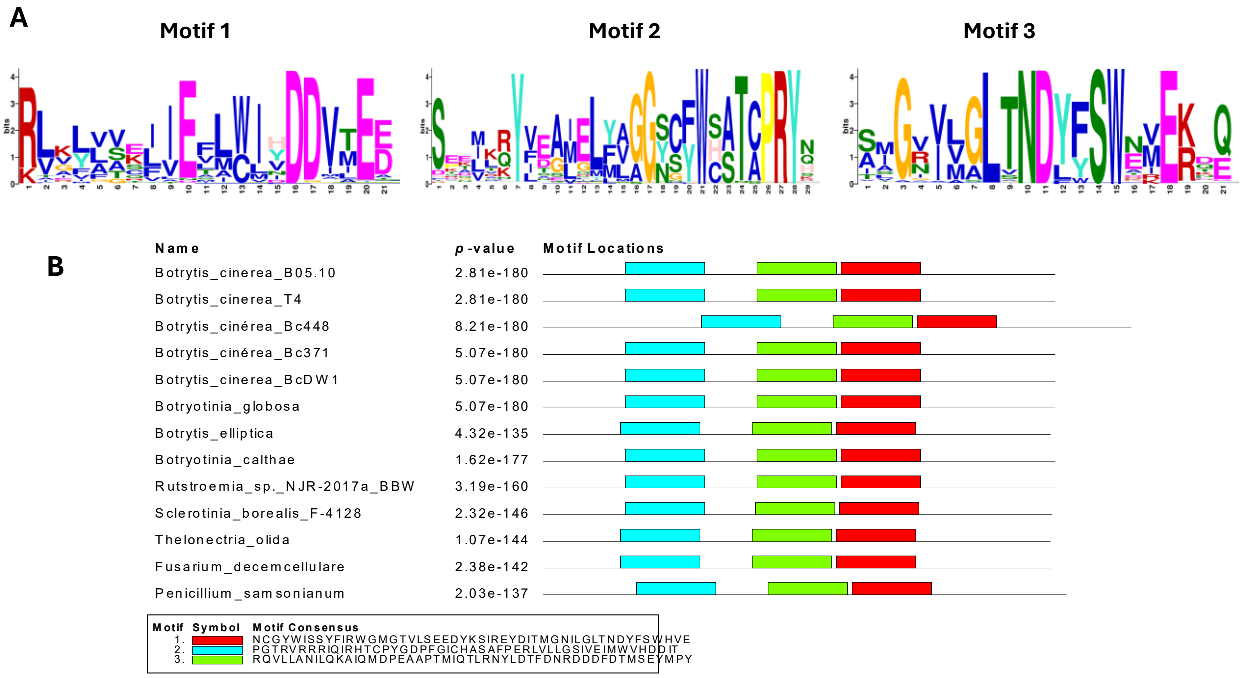
A
Motif 1
Motif 2
Motif 3
B
Name
p-value
Motif Locations
Botrytis_cinerea_B05.10 2.81e-180
Botrytis_cinerea_T4 2.81e-180
Botrytis_cinérea_Bc448 8.21e-180
Botrytis_cinérea_Bc371 5.07e-180
Botrytis_cinerea_BcDW1 5.07e-180
Botryotinia_globosa 5.07e-180
Botrytis_elliptica 4.32e-135
Botryotinia_calthae 1.62e-177
Rutstroemia_sp._NJR-2017a_BBW 3.19e-160
Sclerotinia_borealis_F-4128 2.32e-146
Thelonectria_olida 1.07e-144
Fusarium_decemcellulare 2.38e-142
Penicillium_samsonianum 2.03e-137
Motif Symbol Motif Consensus
1. NCGYWISSYFIRWGMGTVLSEEDYKSIREYDITMGNILGLTNDYFSWHVE
2. PGTRVRRRIQIRHTCPYGDPFGICHASAFPERLVLLGSIVEIMWVHDDIT
3. RQVLLANILQKAIQMDPEAAPTMIQTLRNYLDTFDNRDDDFDTMSEYMPY

Figure S3

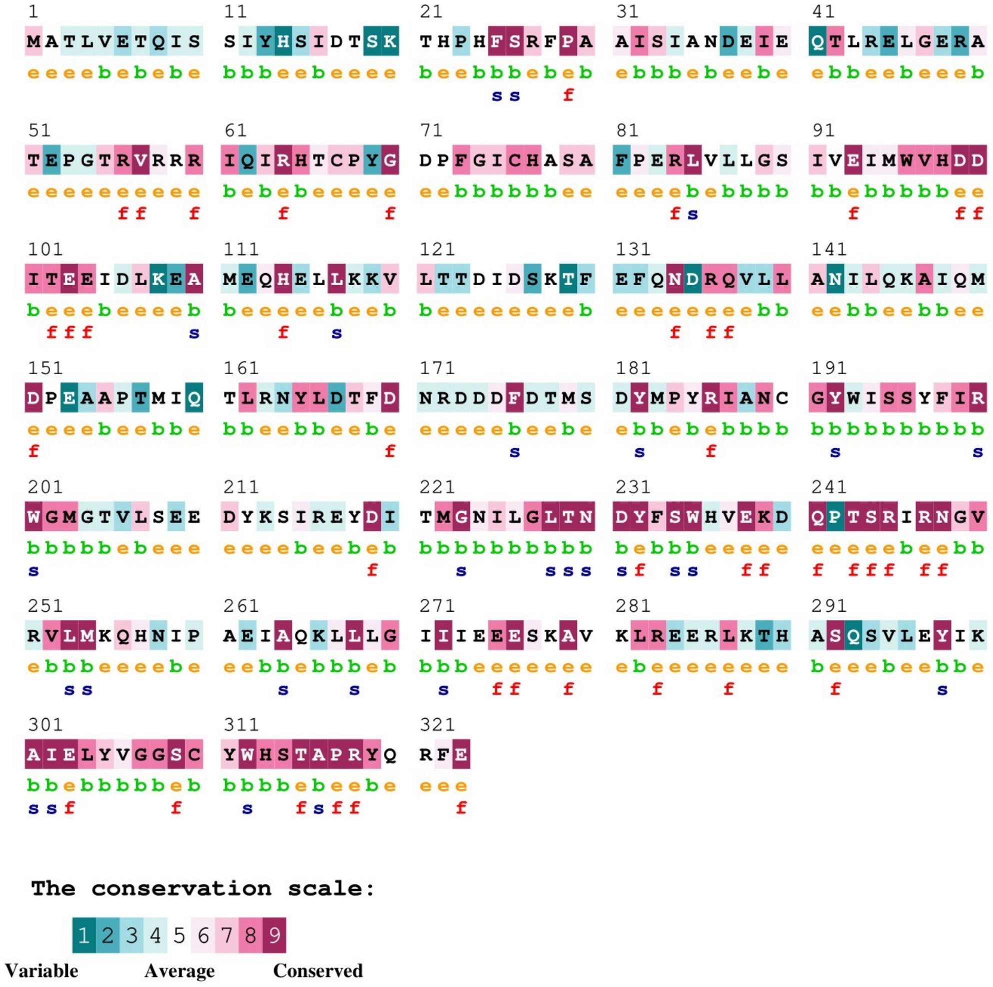

e – An exposed residue according to the neural network algorithm.

b – A buried residue according to the neural network algorithm.

f – A predicted functional residue (highly conserved and exposed).

s – A predicted structural residue (highly conserved and buried).

x – Insufficient data – the calculation for this site was performed on less than 10% of the sequences.

Figure S4

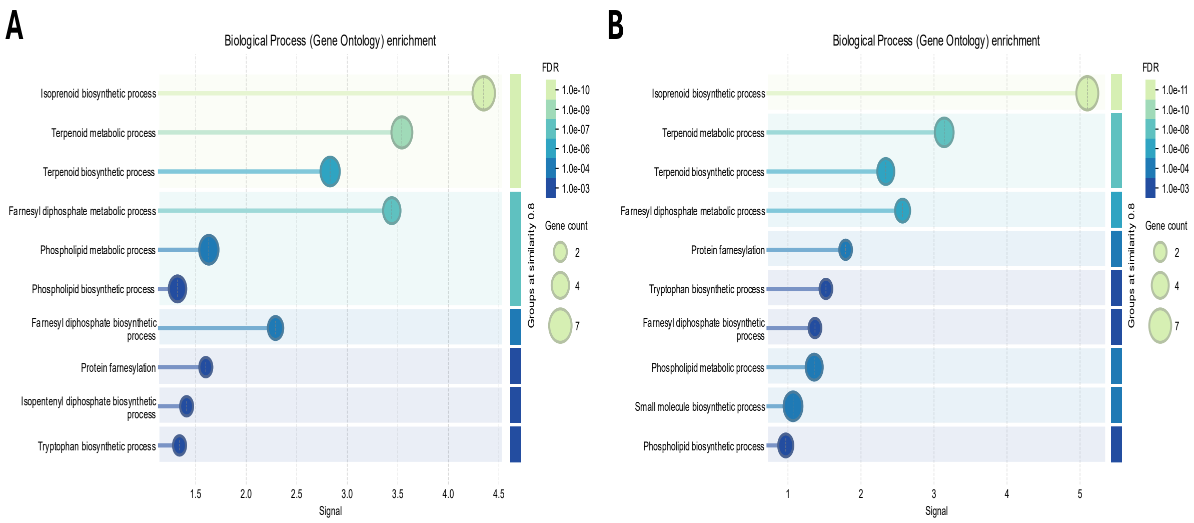

A
Biological Process (Gene Ontology) enrichment
Isoprenoid biosynthetic process
Terpenoid metabolic process
Terpenoid biosynthetic process
Farnesyl diphosphate metabolic process
Phospholipid metabolic process
Phospholipid biosynthetic process
Farnesyl diphosphate biosynthetic process
Protein farnesylation
Isopentenyl diphosphate biosynthetic process
Tryptophan biosynthetic process
1.5
2.0
2.5
3.0
3.5
4.0
4.5
Signal
Groups at similarity 0.8
FDR
1.0e-10
1.0e-09
1.0e-07
1.0e-06
1.0e-04
1.0e-03
Gene count
2
4
7
B
Biological Process (Gene Ontology) enrichment
Isoprenoid biosynthetic process
Terpenoid metabolic process
Terpenoid biosynthetic process
Farnesyl diphosphate metabolic process
Protein farnesylation
Tryptophan biosynthetic process
Farnesyl diphosphate biosynthetic process
Phospholipid metabolic process
Small molecule biosynthetic process
Phospholipid biosynthetic process
1
2
3
4
5
Signal
Groups at similarity 0.8
FDR
1.0e-11
1.0e-10
1.0e-08
1.0e-06
1.0e-04
1.0e-03
Gene count
2
4
7

Figure S5

Enzymatic assay | **Whole cell catalysis**

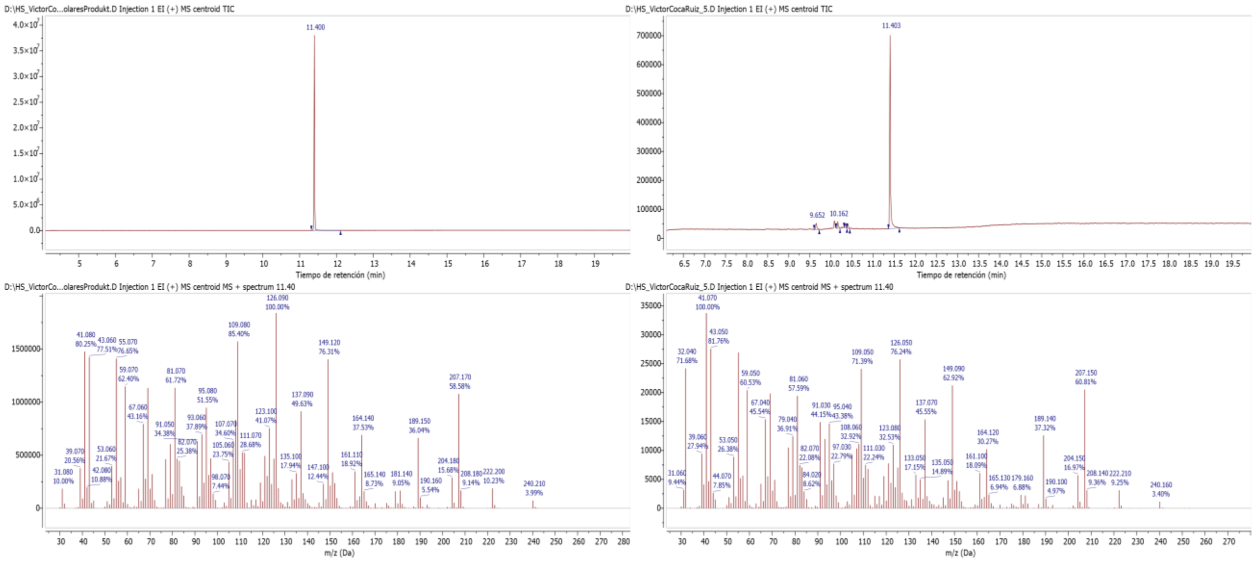

Figure S6

Elemental Composition Report

Single Mass Analysis
Tolerance = 2.0 mDa / DBE: min = -1.5, max = 80.0
Element prediction: Off
Number of isotope peaks used for i-FIT = 3

Monoisotopic Mass, Even Electron Ions
85 formula(e) evaluated with 1 results within limits (up to 5 closest results for each mass)
Elements Used:
C: 0-100 H: 0-200 O: 0-50 Na: 0-1

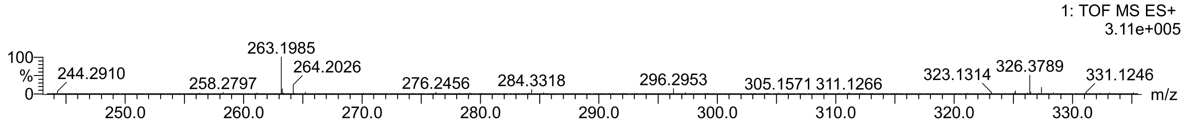

Minimum:				-1.5				
Maximum:		2.0	300.0	80.0				
Mass	Calc. Mass	mDa	PPM	DBE	i-FIT	Norm	Conf(%)	Formula
263.1985	263.1987	-0.2	-0.8	1.5	1029.7	n/a	n/a	C15 H28 O2 Na

Figure S7

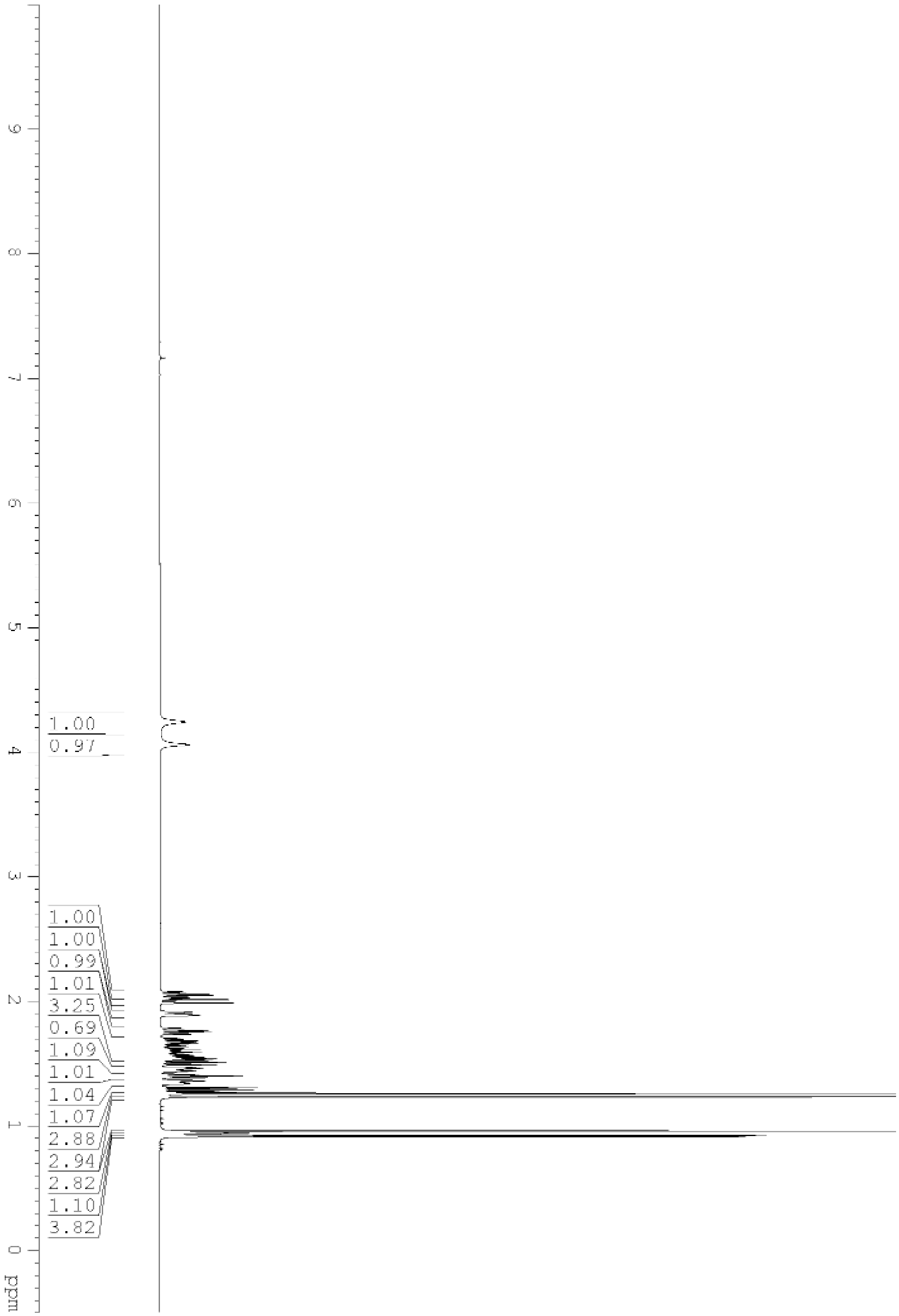

9
8
7
6
5
4
3
2
1
0
ppm
1.00
0.97
1.00
1.00
0.99
1.01
3.25
0.69
1.09
1.01
1.04
1.07
2.88
2.94
2.82
1.10
3.82

Figure S8

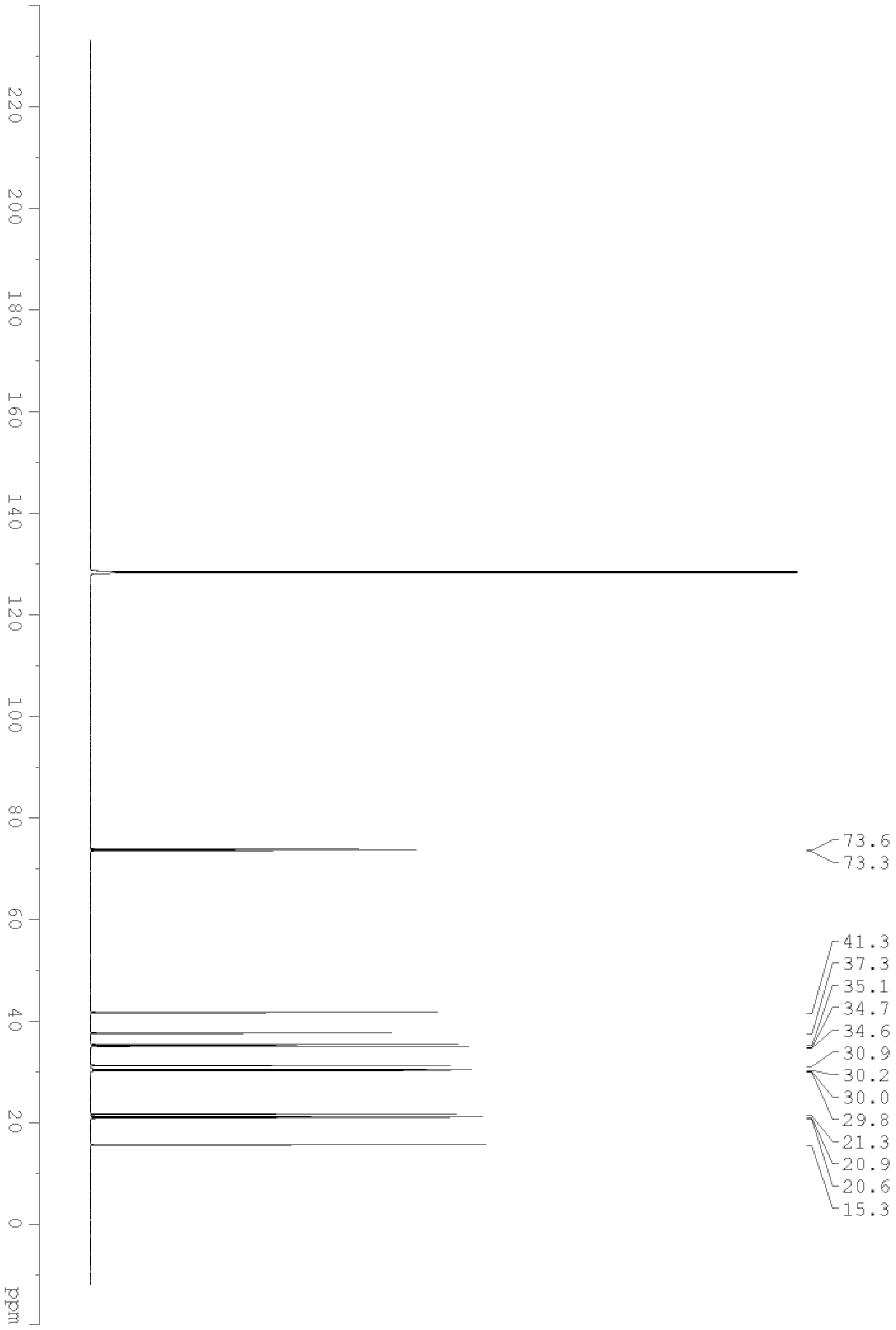

73.6
73.3
41.3
37.3
35.1
34.7
34.6
30.9
30.2
30.0
29.8
21.3
20.9
20.6
15.3
220
200
180
160
140
120
100
80
60
40
20
0
ppm

Figure S9

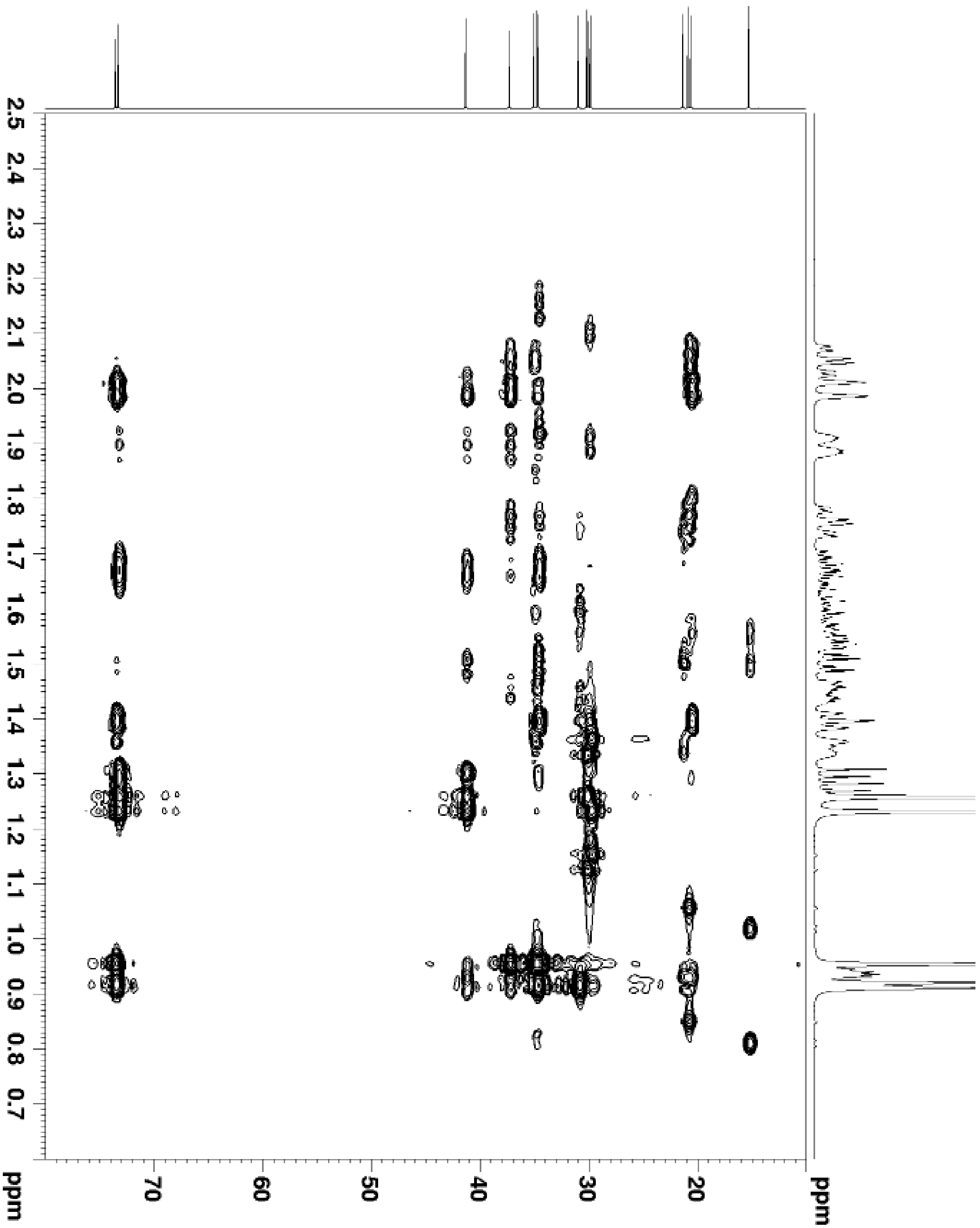

ppm
2.5
2.4
2.3
2.2
2.1
2.0
1.9
1.8
1.7
1.6
1.5
1.4
1.3
1.2
1.1
1.0
0.9
0.8
0.7
ppm
70
60
50
40
30
20

Figure S10

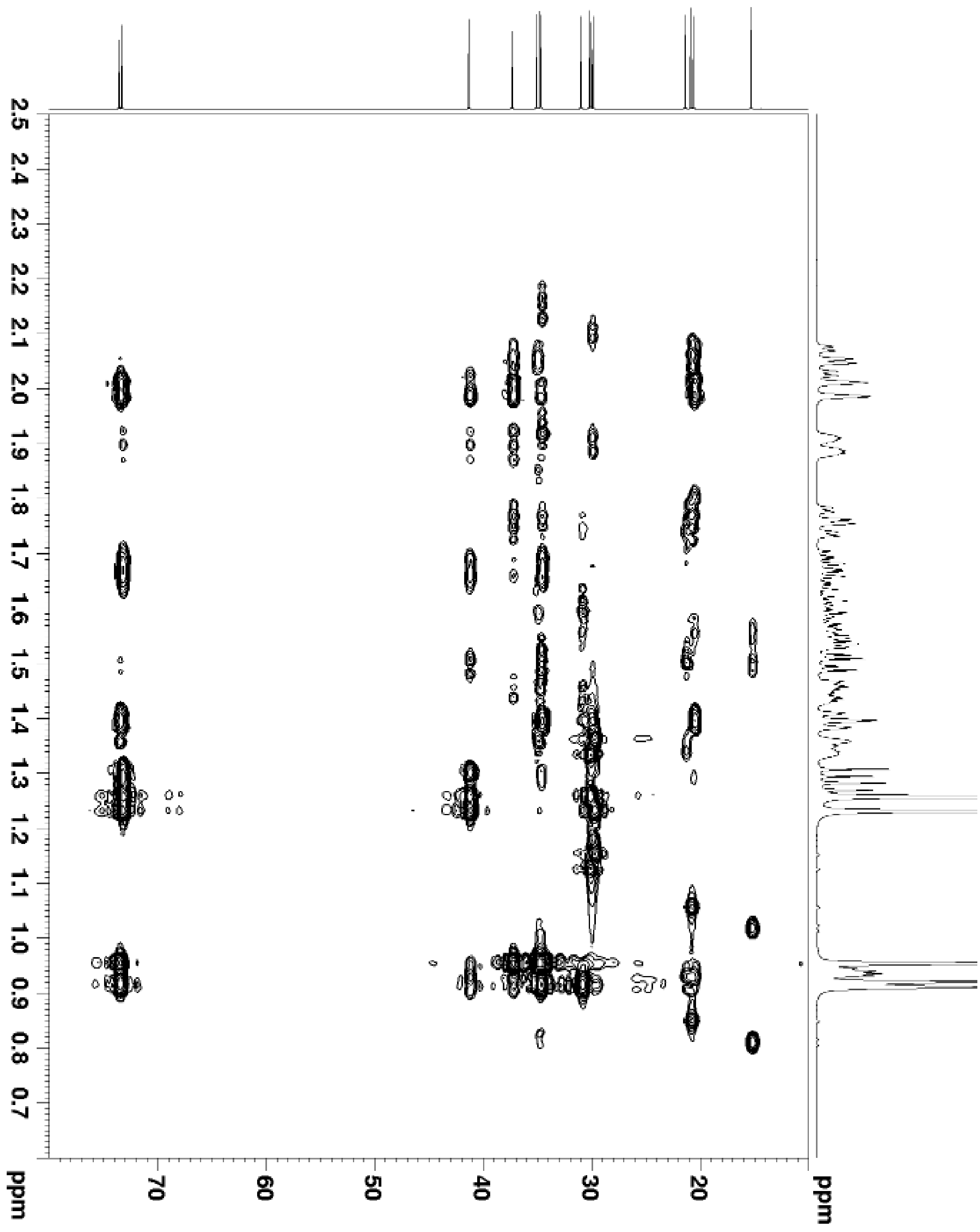

Figure S11

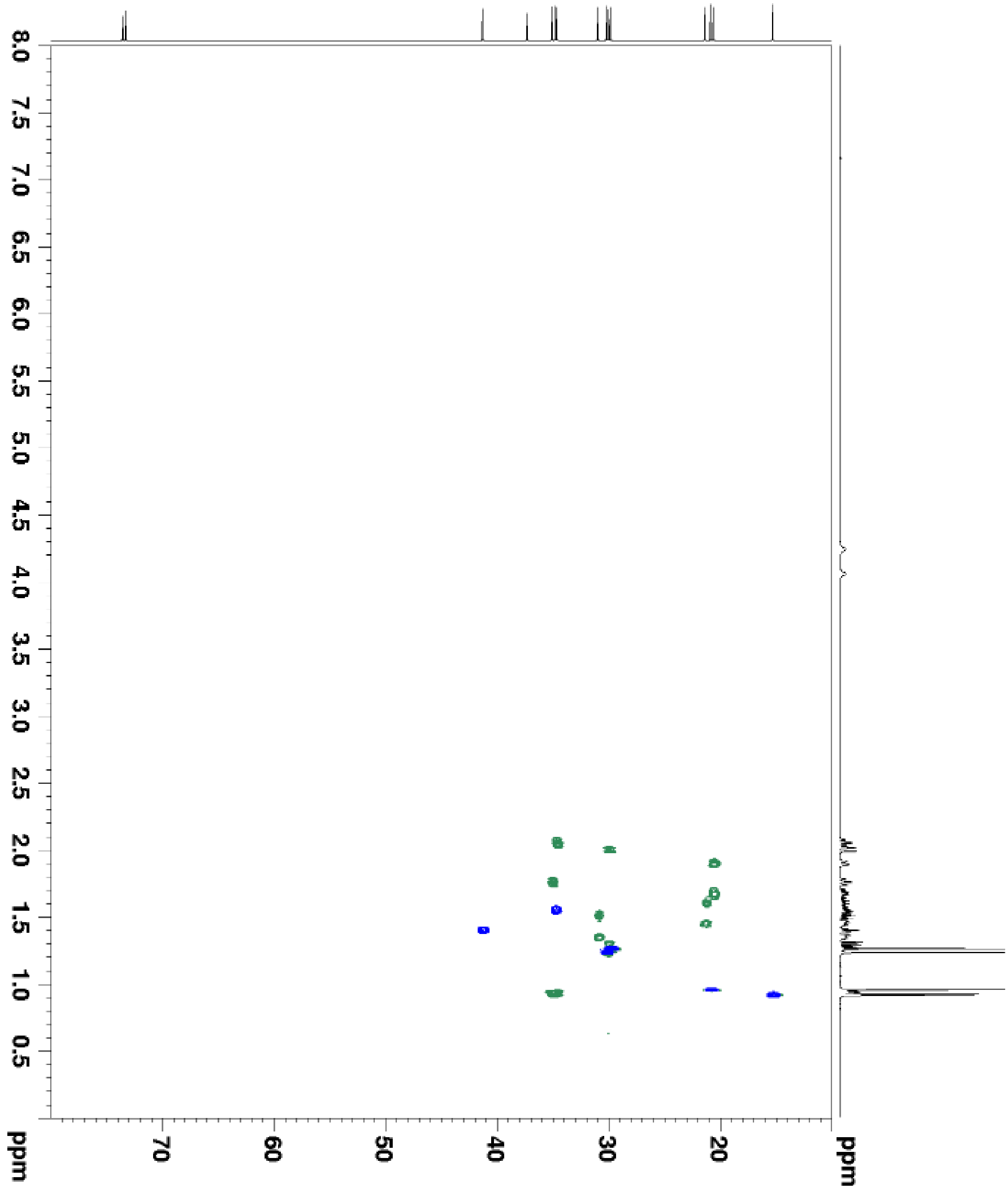

8.0
7.5
7.0
6.5
6.0
5.5
5.0
4.5
4.0
3.5
3.0
2.5
2.0
1.5
1.0
0.5
ppm
70
60
50
40
30
20
ppm

Figure S12

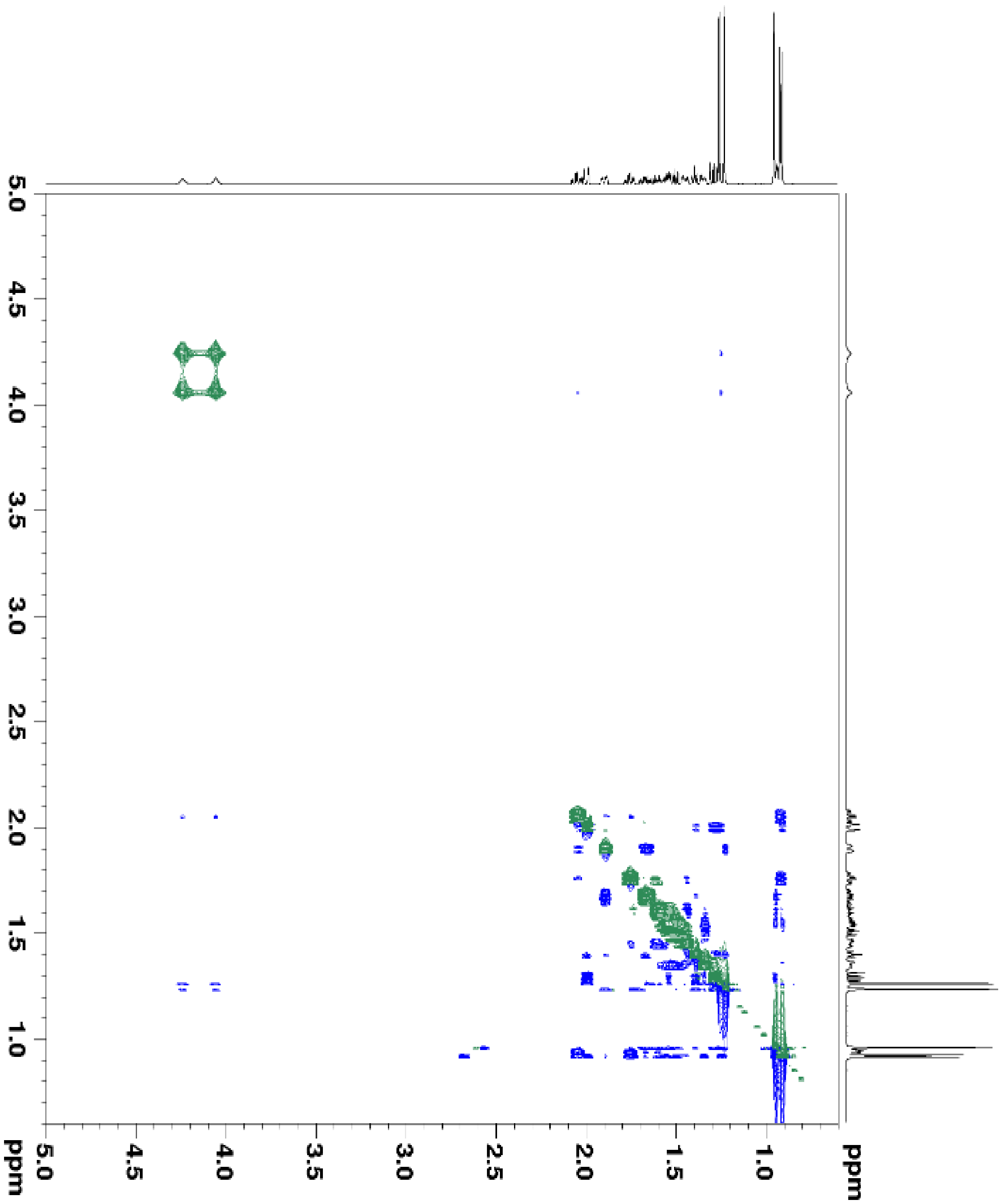

Supplement: Supplementary file 1 [file cb6c00103_si_001.pdf]
